# Transient acceleration of *Lyophyllum decastes* mycelial biomass accumulation by *Acanthopanax senticosus* extract

**DOI:** 10.3389/fmicb.2026.1894250

**Published:** 2026-09-03

**Authors:** Shiyong Wen, Wenjiao Li, Ruizhen Jian, Xunfan Wei, Xiuli Sun, Cheng Liu, Wen Li, Cuiping Wen, Wen Yang, Yifan Yang, Huisong Wang, Zhennan Qiu

**Affiliations:** 1 College of Life Sciences, Dezhou University, Dezhou, Shandong, China; 2 Dezhou Academy of Agricultural Sciences, Dezhou, Shandong, China; 3 Inner Mongolia Academy of Agricultural and Animal Husbandry Sciences, Hohhot, Inner Mongolia, China; 4 Dezhou Municipal Hospital, Dezhou, Shandong, China; 5 Belgorod College of Food Sciences, Dezhou University, Dezhou, Shandong, China

**Keywords:** *Acanthopanax senticosus* aqueous extract, GSEA, *Lyophyllum decastes*, mycelial biomass, submerged cultivation, transcriptome, WGCNA

## Abstract

**Introduction:**

*Lyophyllum decastes* is an edible mushroom with nutritional and medicinal value, but approaches for accelerating mycelial biomass accumulation during submerged cultivation remain limited.

**Methods:**

This study evaluated the effects of Acanthopanax senticosus aqueous extract (ASAE) on submerged mycelial growth and examined associated transcriptional responses using time-course RNA sequencing, weighted gene co-expression network analysis, gene set enrichment analysis, and independent RT-qPCR experiments.

**Results:**

Among the concentrations tested, 50 mg/L ASAE produced the greatest biomass accumulation at day 7, increasing mycelial dry weight by 22.7% relative to the untreated control, whereas 1,000 mg/L ASAE inhibited biomass accumulation. However, the treatment effect was no longer statistically significant at day 9, indicating a transient acceleration of biomass accumulation rather than an increase in final biomass yield under the conditions tested. Two-way ANOVA identified significant effects of cultivation time, treatment, and their interaction on mycelial dry weight. WGCNA module eigengenes were further analyzed using models incorporating cultivation time, treatment, and their interaction. MEblue remained significantly associated with all three factors, including their interaction, but was interpreted as a time- and treatment-associated co-expression module rather than a treatment-specific regulatory module. Direction-separated GSEA showed time-resolved enrichment of gene sets related to energy metabolism, RNA processing, protein homeostasis, carbon metabolism, translation, intracellular trafficking, and carbohydrate-associated metabolism. Upregulated GSEA leading-edge genes were intersected with MEblue genes for candidate-gene visualization without performing a second enrichment analysis. Independent RT-qPCR experiments confirmed the expression trends of selected candidates, including *CHSG*, *UAP1*, *ALG5*, and *RPN12*. A homology-based transcript-collapse sensitivity analysis produced qualitatively similar module- and pathway-level patterns.

**Discussion:**

These findings indicate that ASAE-associated acceleration of *L. decastes* biomass accumulation is accompanied by time-resolved transcriptional remodeling. The proposed biological interpretation remains hypothesis-generating, and direct physiological, biochemical, protein-level, and genetic validation will be required to establish causality.

## 1 Introduction

*Lyophyllum decastes* is an edible and medicinal basidiomycete with increasing commercial interest in East Asian food and functional food markets. Its fruiting bodies contain proteins, essential amino acids, and bioactive polysaccharides, particularly β-glucans, which have been associated with immunomodulatory, antitumor, antidiabetic, hypolipidemic, and gut microbiota-modulating activities (Miura et al., 2002; Wasser, 2002; Kokean et al., 2005; Ding et al., 2022; Zhang et al., 2023). Recent genomic and transcriptomic studies have provided insights into fruiting-body development, phylogenetic relationships, and lignocellulose-degrading potential in *L. decastes* and related species (Ke et al., 2023; Xu et al., 2023). In practice, however, production of *L. decastes* still largely depends on conventional fruiting-body cultivation, which is time-consuming and can be influenced by substrate composition, environmental conditions, and cultivation management (Wei et al., 2006; Xu et al., 2023).

Submerged cultivation provides a practical alternative for producing edible fungal mycelia and associated metabolites without completing the full fruiting-body stage. Compared with solid-state cultivation, liquid culture allows tighter control of medium composition, aeration, agitation, and culture duration, which is useful for process optimization and scale-up. In *L. decastes*, submerged culture has been investigated for mycelial yield and polysaccharide production, and mycelial cultures have also been used to develop fermented products such as soybean milk- and corn juice-based beverages (Pokhrel and Ohga, 2007; Zhang et al., 2013). Nevertheless, biomass production in liquid culture remains limited by the relatively slow growth of *L. decastes* mycelia, and the transcriptional events associated with biomass accumulation in this cultivation system are still insufficiently defined.

Plant-derived hot-water extracts contain complex mixtures of nutritional and non-nutritional constituents and may influence fungal growth in ways that are not limited to carbon supplementation. Components such as soluble polysaccharides, phenolics, glycosides, lignans, minerals, and other small molecules may affect mycelial growth, pellet morphology, stress adaptation, or metabolite accumulation depending on fungal species and culture conditions. *Acanthopanax senticosus* is a food-associated medicinal plant rich in polysaccharides and phenolic glycosides, including syringin and eleutheroside E (Davydov and Krikorian, 2000; Huang et al., 2011; Jia et al., 2021). A previous study reported that extracts from Chinese herbs influenced active compound formation during liquid fermentation of *Auricularia auricula* (Xu et al., 2020), suggesting that plant-derived extracts can modulate edible fungal fermentation. Whether *A. senticosus* aqueous extract can promote *L. decastes* mycelial biomass production, however, remains unclear. It is also unknown whether such effects are associated with stage-specific transcriptional responses during submerged cultivation.

Mycelial biomass accumulation is a complex biological process involving coordinated hyphal extension, branching, pellet formation, cell-wall expansion, membrane synthesis, energy production, intracellular trafficking, protein synthesis, and stress adaptation. Polarized hyphal growth requires continuous vesicle trafficking, cytoskeleton-associated transport, and cell-wall remodeling at the growing apex (Steinberg, 2007; Riquelme et al., 2011; Free, 2013). The fungal cell wall is a dynamic structure closely associated with growth, morphogenesis, environmental adaptation, and cellular integrity (Free, 2013; Hopke et al., 2018). In parallel, energy-generating pathways provide ATP and reducing power required for biosynthesis, vesicle trafficking, and active transport. RNA processing, ribosome biogenesis, protein folding, glycosylation, and proteasome-mediated quality control also support the synthesis, maturation, and maintenance of enzymes and structural proteins required for fungal growth (Hartl et al., 2011; Balchin et al., 2016). Therefore, a time-resolved transcriptomic approach can help reveal how plant-derived aqueous extracts influence the coordinated molecular processes associated with submerged mycelial biomass accumulation.

In this study, we evaluated the concentration-dependent effects of ASAE on *L. decastes* mycelial biomass accumulation during submerged cultivation and then performed time-course RNA sequencing to characterize ASAE-associated transcriptional responses. Weighted gene co-expression network analysis, gene set enrichment analysis, functional enrichment, and RT-qPCR validation were integrated to identify biomass-associated gene modules and stage-dependent candidate pathways. By linking growth phenotypes with transcriptional signatures, this study aimed to clarify the molecular processes associated with ASAE-enhanced mycelial biomass accumulation and to provide candidate pathways and genes for future functional validation and process optimization.

## 2 Materials and methods

### 2.1 Isolation and molecular identification of the *Lyophyllum decastes* strain

Fresh fruiting bodies of the industrially cultivated edible mushroom commercially known as *Lyophyllum decastes* were obtained from Dezhou Xinsheng Mycological Technology Co., Ltd., (Shandong, China) through a local supermarket. Fruiting bodies without visible contamination or mechanical damage were stored at 4 °C and processed within 24 h. For tissue isolation, the fruiting bodies were surface-sterilized with 75% ethanol for 30 s, rinsed three times with sterile distilled water, and dissected under aseptic conditions. Small internal tissue blocks were excised from the middle-to-lower region of the stipe and inoculated onto potato dextrose agar (PDA; Hopebio, Qingdao, China) slants in sterile test tubes. The slants were incubated at 25 °C in darkness until mycelia emerged from the tissue blocks. Hyphal tips from actively growing colony margins were transferred twice onto fresh PDA slants to obtain a purified culture. The resulting isolate was designated LD-DZ2025 and maintained on PDA slants at 4 °C as the working culture. Mycelial plugs were preserved in 20% glycerol at −80°C for long-term storage.

Freeze-dried mycelia of LD-DZ2025 were used for genomic DNA extraction with the Fungal Genomic DNA Rapid Extraction Kit (Sangon Biotech, Shanghai, China; Cat. No. B518229). After extraction, the DNA preparation was incubated with RNase A (Thermo Fisher Scientific; Cat. No. EN0531) at 37 °C for 30 min to remove residual RNA. The internal transcribed spacer (ITS) region was amplified using ITS1, 5′-TCCGTAGGTGAACCTGCGG-3′, and ITS4, 5′-TCCTCCGCTTATTGATATGC-3′. The thermal cycling conditions were 95 °C for 1 min; 35 cycles of 95 °C for 30 s, 55 °C for 30 s, and 72 °C for 30 s; followed by 72 °C for 3 min. PCR products were examined on agarose gels before bidirectional sequencing at Sangon Biotech Co., Ltd. (Shanghai, China). Forward and reverse chromatograms were assembled and manually checked to obtain a consensus ITS sequence. The sequence was compared with entries in the NCBI GenBank nucleotide database using BLASTn. Representative ITS sequences from *Lyophyllum* species and related taxa were then used to evaluate the phylogenetic position of LD-DZ2025, with *L. decastes* HM572548.1 included as the reference sequence. Multiple sequence alignment and maximum-likelihood phylogenetic reconstruction were performed in MEGA X (Kumar et al., 2018). The substitution model was selected based on the Bayesian information criterion, and branch support was assessed with 1,000 bootstrap replicates.

### 2.2 Preparation of liquid inoculum and ASAE

For liquid inoculum preparation, LD-DZ2025 was cultured on PDA slants in sterile test tubes at 25 °C in darkness for 15 days, until the slant surface was fully covered by mycelia. The entire mycelial mat from each slant was scraped aseptically and transferred into a 250-mL Erlenmeyer flask containing 100 mL of sterile potato dextrose broth (PDB) and a Teflon-coated magnetic stir bar. The flasks were incubated at 25 °C in darkness on a rotary shaker at 120 rpm. After pellet-like aggregates had formed by day 7, magnetic stirring was applied to disperse the pellets, and the cultures were maintained until day 10 to obtain a flocculent mycelial suspension. Before inoculation into the formal experiments, the suspension was gently mixed under aseptic conditions. Magnetic stirring was used only during preparation of the liquid inoculum and was not applied during ASAE treatment cultures. As an additional check of cultivation consistency, the preserved LD-DZ2025 culture was returned to Dezhou Xinsheng Mycological Technology Co., Ltd., and grown under standard industrial production conditions. The fruiting bodies obtained from this test were compared visually with the company’s routinely cultivated *L. decastes*.

Dried bark of *Acanthopanax senticosus* was purchased as 40-mesh powder from Jilin Lishengyuan Biological Products Co., Ltd. (Jilin, China). Briefly, 100 g of dried bark powder was extracted twice with 500 mL of boiling distilled water at 100 °C for 30 min per extraction using a reflux condenser. The combined extracts were filtered through sterile gauze and filter paper to remove insoluble residues, and the filtrate was vacuum-concentrated to 100 mL at 40 °C and 0.09 MPa using a DLAB RE100-Pro rotary evaporator. The concentrate was frozen at −80°C for 12 h and lyophilized for 48 h using a SCIENTZ-10ND/A freeze dryer to obtain crude ASAE powder. ASAE powder was stored at −20 °C in airtight containers protected from light and moisture. Before each experiment, ASAE powder was dissolved in sterile distilled water to prepare stock solutions. The solutions were passed through a 0.22-μm membrane filter under aseptic conditions and then added to sterile PDB medium to reach the required final ASAE concentrations. Total polysaccharides in ASAE were measured by the phenol–sulfuric acid assay, using glucose to generate the standard curve (Dubois et al., 1956). Absorbance was recorded at 490 nm, and polysaccharide content was expressed as a percentage of ASAE dry weight. Syringin and eleutheroside E were analyzed by HPLC on an Agilent 1260 Infinity II system fitted with a Zorbax Eclipse Plus C18 column, 4.6 × 250 mm, 5 μm. The mobile phase contained 0.1% formic acid in water as solvent A and acetonitrile as solvent B. Compounds were eluted with a linear gradient from 10% to 30% B over 20 min at a flow rate of 1.0 mL/min and detected at 207 nm. Quantification was performed against authentic syringin and eleutheroside E standards from Sigma-Aldrich, both with purity ≥98%, Cat. Nos. SML1767 and E9407, respectively.

### 2.3 ASAE treatment experiments and time-course sampling

A concentration-screening experiment was first carried out to determine how ASAE affected *L. decastes* mycelial growth. For each culture, 5 mL of well-mixed liquid inoculum was added aseptically to a 250-mL Erlenmeyer flask containing 100 mL of sterile PDB medium. ASAE was supplied at final concentrations of 0, 1, 5, 10, 50, 100, 500, and 1,000 mg/L. The 0 mg/L treatment contained the same volume of sterile distilled water and was used as the untreated control. Three independently inoculated flasks were prepared for each concentration. Cultures were grown at 25 °C in the dark on a rotary shaker at 120 rpm for 7 days. During ASAE treatment, mycelial pellets were not dispersed by magnetic stirring or other mechanical disruption before biomass measurement or RNA sampling.

After 7 days, mycelia were collected by centrifugation at 8,000 × *g* for 10 min at 4 °C in an Eppendorf 5910 R centrifuge. The pellets were washed twice with sterile RNase-free water to remove residual medium and ASAE. For biomass measurement, the washed mycelia were freeze-dried for 24 h at −80 °C and 0.01 mbar until constant weight and then weighed on an analytical balance. Dry weight was recorded separately for each biological replicate.

Based on the biomass response observed in the concentration-screening experiment, 50 mg/L ASAE was chosen for the subsequent time-course transcriptome analysis. The sampling scheme included a shared day-0 baseline collected before ASAE addition and six later groups collected at days 3, 5, and 7. The day-0 baseline was designated B0. Untreated cultures were designated B3, B5, and B7 according to the sampling day, and ASAE-treated cultures were designated T3, T5, and T7. Three biological replicates were prepared for each group, giving 21 RNA-seq samples in total. Each replicate was derived from an independently inoculated flask. At each sampling time, mycelia were harvested promptly, washed with RNase-free water, blotted to remove excess liquid, frozen in liquid nitrogen, and kept at −80°C until RNA extraction. For each RNA-seq sample, mycelial dry weight was measured in parallel, allowing biomass measurements to be paired with the corresponding gene-expression profiles in the module–trait correlation analysis.

To quantitatively assess the visually observed differences in pellet coverage, original images from three independent cultivation batches were analyzed using a blinded two-dimensional image-analysis procedure at days 7. The analyst was unaware of treatment identity during image processing. The proportion of the culture area occupied by visible mycelial pellets was calculated as pellet coverage. Coverage values were summarized for each independent batch, and the paired Control–ASAE differences were evaluated using a two-tailed paired *t*-test.

### 2.4 RNA isolation, library preparation, and sequencing

Total RNA was extracted from frozen mycelial samples using TRIzol™ reagent, Sangon Biotech, Cat. No. B511311, according to the manufacturer’s protocol. To minimize genomic DNA contamination, RNA samples were further treated with on-column DNase I digestion, Qiagen, Cat. No. 79254. RNA concentration and purity were evaluated using a NanoDrop™ 2000 spectrophotometer, Thermo Fisher Scientific, and RNA integrity was assessed using an Agilent 2100 Bioanalyzer, Agilent Technologies. Only RNA samples with OD260/280 values between 1.8 and 2.2 and RNA integrity number, RIN, values ≥8.0 were used for library construction.

For each sample, 1 μg of total RNA was used for stranded mRNA library preparation. Polyadenylated mRNA was enriched using Dynabeads Oligo(dT)25, Thermo Fisher Scientific, Cat. No. 25-61005, and fragmented to approximately 300 bp using the NEBNext^®^ Magnesium RNA Fragmentation Module at 94 °C for 5 min. cDNA synthesis, end repair, adaptor ligation, and PCR enrichment were performed using the Illumina TruSeq™ Stranded mRNA Library Prep Kit according to the manufacturer’s instructions. Libraries were size-selected to approximately 200–500 bp using AMPure XP beads and quality-checked by qPCR and fragment-size analysis.

Qualified libraries were sequenced on an Illumina NovaSeq 6000 platform using a paired-end 150 bp sequencing strategy. Approximately 20 million paired-end reads were generated per sample.

### 2.5 RNA-seq quality control, *de novo* transcriptome assembly, and functional annotation

Raw paired-end reads were processed using fastp v0.23.2 to remove adaptor sequences, reads containing excessive ambiguous bases, and low-quality reads (Chen et al., 2018). Quality trimming was performed using a sliding-window approach with a window size of 4 bp, and low-quality reads were filtered according to the default quality-control criteria. The quality of clean reads was assessed based on Q20, Q30, GC content, clean-read ratio, and sequencing depth. Clean reads from all 21 samples were used for downstream transcriptome assembly and expression analysis.

Although genome sequence information for *L. decastes* has recently become available (Xu et al., 2023), comprehensive and publicly accessible structural gene annotation remains limited. Therefore, a *de novo* transcriptome assembly strategy was adopted to reconstruct expressed transcripts in the present study. High-quality clean reads were assembled using Trinity v2.15.1 with default parameters unless otherwise specified, including k-mer size 25 and a minimum contig length of 200 bp (Grabherr et al., 2011). To reduce redundancy, assembled transcripts were clustered using CD-HIT-EST v4.8.1 with a sequence identity threshold of 95% and word size of 5 (Fu et al., 2012). The longest representative sequence from each cluster was retained as a unigene for subsequent annotation and expression analysis. Transcript and unigene assembly statistics, including total number, total length, N50, mean length, and maximum length, were calculated and summarized.

Assembly quality was evaluated using TransRate v1.0.3 and BUSCO v5.4.3 (Smith-Unna et al., 2016; Manni et al., 2021). BUSCO analysis was conducted using the fungi_odb10 lineage dataset, consistent with the assembly quality assessment reported in Supplementary Table 2. The proportions of complete single-copy, complete duplicated, fragmented, and missing BUSCOs were reported. Because *de novo* transcriptome assemblies often contain isoform redundancy and closely related transcript variants, duplicated BUSCOs were interpreted as an indicator of transcript-level redundancy rather than direct evidence of genome-level gene duplication.

Functional annotation of unigenes was performed by sequence similarity searches against public databases. DIAMOND v2.1.6 blastx was used to search against the NCBI non-redundant protein (NR), Swiss-Prot, and KEGG databases with an *E*-value cutoff of 1 × 10^−5^. Protein domain annotation was performed against the Pfam database using HMMER. Orthologous group assignment was conducted using eggNOG and COG/KOG annotations where applicable. Gene Ontology (GO) terms were assigned based on database matches and domain annotations (Subramanian et al., 2005). Annotation results from NR, Swiss-Prot, Pfam, eggNOG, KEGG, and GO were integrated to generate the final functional annotation table. For expression quantification, clean reads from each sample were mapped back to the representative unigene set, and unigene-level expression abundance was estimated using RSEM (Li and Dewey, 2011) within the standard RNA-seq workflow implemented on the Majorbio Cloud Platform. Expression levels were normalized and reported as transcripts per million (TPM). The resulting unigene-level TPM matrix was used for sample-level quality assessment, including pairwise Pearson correlation analysis and principal component analysis (PCA), using R v4.3.2.

### 2.6 Transcript-collapse sensitivity analysis

To assess the potential influence of transcript redundancy in the *de novo* assembly, a homology-based transcript-collapse sensitivity analysis was performed. Expressed unigenes were grouped into orthologue-level units using eggNOG orthogroup annotations, followed by KO and Swiss-Prot homolog annotations. When multiple unigenes were assigned to the same orthologue-level unit, the unigene with the highest mean expression across all samples was retained as the representative transcript. The collapsed expression matrix was analyzed using the same WGCNA and direction-separated GSEA procedures as the original unigene-level matrix. This analysis was used to evaluate whether the principal module-level and enrichment-based conclusions were qualitatively robust to transcript collapse in the absence of a species-specific reference genome.

### 2.7 Weighted gene co-expression network analysis and direction-separated gene set enrichment analysis

WGCNA was performed on the Majorbio Cloud Platform. Unigene-level TPM values from all 21 RNA-seq samples were used after filtering unigenes with mean TPM < 1 or coefficient of variation <0.1. After sample clustering confirmed the absence of obvious outliers, a signed hybrid network was constructed using a soft-thresholding power of β = 16. Modules were detected from the TOM-based dissimilarity matrix using dynamicTreeCut v1.63 (Langfelder et al., 2008) with a minimum module size of 30 and deepSplit = 2. Module eigengenes (MEs) were calculated as the first principal component of each module. Because cultivation time and ASAE treatment were both associated with large-scale variation in gene expression, module eigengenes were analyzed using linear models containing cultivation time, treatment, and their interaction. The model was specified as:


ME∼time+treatment+time:treatment

For the biomass trait, module–trait associations were also assessed using models containing mycelial dry weight together with cultivation time and treatment. Accordingly, MEblue was interpreted as a module associated with time, treatment, and their interaction, rather than as a treatment-specific regulatory module. Gene significance and module membership were calculated for descriptive prioritization of candidate genes.

GSEA was performed using GSEA v4.3.2 for the T3 versus B3, T5 versus B5, and T7 versus B7 comparisons. All expressed unigenes were ranked using the Signal2Noise metric without applying a fixed fold-change threshold. GO and KEGG gene sets were constructed from the annotation results generated in this study, and expressed annotated unigenes were used as the background gene universe. Gene sets containing 15–500 genes were retained. Significant enrichment was defined as an FDR-adjusted *p*-value < 0.05 and | NES| > 1. Positive and negative enrichment results were analyzed separately. A positive NES indicated enrichment toward ASAE-treated samples, whereas a negative NES indicated enrichment toward untreated control samples. Leading-edge genes from positive and negative gene sets were not pooled. The complete direction-separated GSEA results, including gene-set name, description, group, gene-set size, ES, NES, nominal *p*-value, adjusted *p*-value, rank at maximum, and leading-edge genes, are provided in Supplementary Table 7.

### 2.8 Integrative WGCNA–GSEA analysis and candidate-gene visualization

To identify candidate genes associated with time-resolved ASAE responses, MEblue genes were intersected separately with leading-edge genes from positively enriched GSEA gene sets at days 3, 5, and 7. The resulting intersections were used to visualize genes that were jointly associated with the biomass-related co-expression module and positive ASAE-associated pathway enrichment. Because the leading-edge genes were selected partly on the basis of GO or KEGG gene-set membership, the intersected genes were not subjected to a second GO or KEGG over-representation analysis. Instead, the original direction-separated GSEA results were presented directly, and the WGCNA–GSEA intersections were used for candidate-gene and pathway-linked visualization in Figures 4, 5. The resulting associations were interpreted as hypothesis-generating and were not considered independent enrichment evidence.

### 2.9 Real-time quantitative PCR validation

Total RNA extraction and quality control were performed as described in section “2.4 RNA isolation, library preparation, and sequencing.” First-strand cDNA was synthesized from 1 μg of total RNA using TransScript^®^ One-Step gDNA Removal and cDNA Synthesis SuperMix, TransGen Biotech, Beijing, China, according to the manufacturer’s protocol. Seven candidate reference genes, including α-tubulin, β-actin, GAPDH, ACLY, RPL3, RPL7, and V-ATP, were evaluated for expression stability using RefFinder, which integrates geNorm, NormFinder, BestKeeper, and the comparative ΔCt method (Vandesompele et al., 2002; Andersen et al., 2004; Pfaffl et al., 2004; Xie et al., 2023). Based on the comprehensive stability ranking and geNorm analysis, α-tubulin and GAPDH were selected as internal reference genes, and the geometric mean of their expression levels was used for normalization.

Primer sequences for the candidate reference and target genes are provided in Supplementary Table 10. Primer specificity was confirmed by melting-curve analysis, and only primer pairs producing a single amplification peak were used. Amplification efficiencies were determined from standard curves generated using serially diluted cDNA; primer pairs with R^2^ ≥ 0.990 and efficiencies of 98%–103% were considered acceptable. RT-qPCR reactions were performed on a LightCycler^®^ 96 Instrument (Roche Diagnostics, Basel, Switzerland) using 2 × PerfectStart™ Green qPCR SuperMix (TransGen Biotech, Beijing, China). The thermal cycling program consisted of initial denaturation at 95 °C for 20 s, followed by 40 cycles of 95°C for 15 s and 60°C for 20 s. Independent RT-qPCR experiments were performed using newly prepared biological samples rather than RNA aliquots from the RNA-seq libraries. Three independent biological replicates were analyzed for each treatment and sampling time, with three technical replicates per biological sample. Relative expression levels were calculated using the 2^−ΔΔCt method (Livak and Schmittgen, 2001). For treatment comparisons, the corresponding untreated control at the same time point was used as the calibrator; for temporal expression analysis, the day-3 untreated control was used as the baseline calibrator when indicated in the figure legends. The RT-qPCR experiments were designed and reported in accordance with the MIQE guidelines (Bustin et al., 2025). Raw Ct values, relative-expression values, individual biological replicate values, primer efficiencies, and amplification statistics are provided in Supplementary Table 10. The consistency between RNA-seq and RT-qPCR results was evaluated by Pearson correlation analysis of log2 fold-change values across the 21 gene-time-point combinations. Because the candidate genes were selected from the transcriptome-derived candidate set, the independent RT-qPCR experiments were used to confirm the direction and temporal trends of selected expression changes; they did not constitute independent validation of pathway activity, WGCNA module function, or causal involvement of individual genes in biomass accumulation.

### 2.10 Statistical analysis

Statistical analyses were performed using GraphPad Prism v10.1.2 (GraphPad Software, San Diego, CA, USA) and R v4.3.2. Phenotypic and RT-qPCR data from three independent biological replicates are presented as mean ± standard deviation (SD). Normality and homogeneity of variance were assessed using the Shapiro–Wilk and Brown–Forsythe tests, respectively. Multiple-group comparisons were analyzed by one-way or two-way ANOVA followed by Tukey’s HSD or Šidák’s multiple comparisons tests, as appropriate. Pearson correlation analysis was used to compare RNA-seq- and RT-qPCR-derived log2 fold-change values. For transcriptome-wide analyses, multiple testing correction was performed using the false discovery rate (FDR) or Benjamini–Hochberg adjusted *p*-values as specified above. Statistical significance was defined at *p* < 0.05, with *, **, and *** indicating *p* < 0.05, *p* < 0.01, and *p* < 0.001, respectively.

## 3 Results

### 3.1 Identification of isolate LD-DZ2025 and chemical characterization of ASAE

**FIGURE 1:**
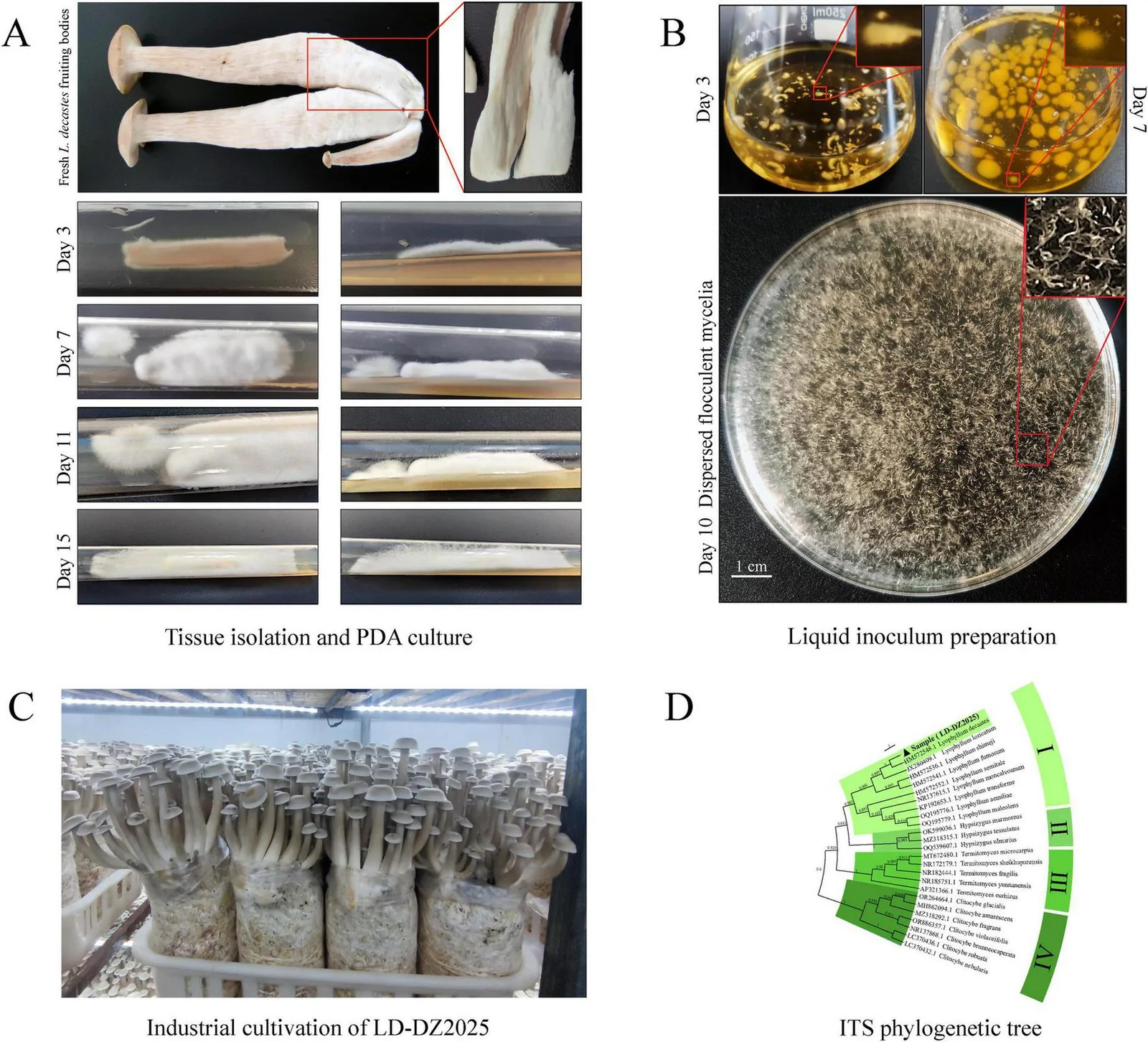
Isolation, cultivation performance, and ITS-based identification of *Lyophyllum decastes isolate LD-DZ2025.* **(A)** Tissue isolation and mycelial development of LD-DZ2025 on PDA slants at 25 °C. Mycelia emerged after 3 days and covered the slant by day 15. **(B)** Liquid inoculum preparation in PDB medium. Pellet-like aggregates formed by day 7 and were dispersed by magnetic stirring to obtain a flocculent mycelial suspension by day 10. Magnetic stirring was not applied during the formal ASAE treatment experiments. Scale bar = 1 cm. **(C)** Fruiting bodies produced from the preserved LD-DZ2025 culture under standard industrial cultivation conditions. **(D)** Maximum-likelihood phylogenetic tree based on ITS sequences. LD-DZ2025 is marked with a black triangle. Bootstrap values from 1,000 replicates are shown at nodes. The scale bar represents substitutions per site.

The fungal isolate LD-DZ2025 was obtained by tissue isolation from fresh fruiting bodies of the industrially cultivated edible mushroom commercially known as *L. decastes*. Internal stipe tissue blocks cultured on PDA slants in sterile test tubes produced visible mycelial outgrowth after 3 days and developed dense, cottony white mycelia that covered the slant medium by day 15 at 25 °C (Figure 1A). During liquid inoculum preparation, all mycelia from each 15-day PDA slant were scraped and transferred into PDB. Mycelial outgrowth and early proliferation were observed after 3 days of liquid cultivation, pellet-like aggregates formed by day 7, and magnetic stirring initiated on day 7 dispersed the pellets to generate a flocculent mycelial suspension by day 10 (Figure 1B). After the preserved LD-DZ2025 culture was returned to Dezhou Xinsheng Mycological Technology Co., Ltd., for industrial cultivation under standard production conditions, the resulting fruiting bodies were visually comparable to the company’s routinely cultivated *L. decastes* fruiting bodies (Figure 1C). This cultivation test supported the cultivation consistency of LD-DZ2025 but was not used as an independent taxonomic criterion.

Internal transcribed spacer sequencing generated high-quality bidirectional reads for LD-DZ2025 (Supplementary Figures 1, 2). The assembled consensus ITS sequence showed 100% identity and full query coverage to the reference *L. decastes* sequence HM572548.1 in GenBank. Phylogenetic analysis placed LD-DZ2025 within the *Lyophyllum* clade containing the reference *L. decastes* sequence HM572548.1 and closely related species, including *L. loricatum* JX280409.1, *L. shimeji* HM572536.1, and *L. fumosum* HM572541.1 (Figure 1D). Thus, LD-DZ2025 was assigned to cultivated *L. decastes* based on combined evidence from source information, morphology, ITS sequence similarity, phylogenetic placement, and cultivation performance, although ITS-based resolution among closely related *Lyophyllum* species remains limited. ASAE was prepared by hot-water extraction from *Acanthopanax senticosus* bark. Total polysaccharides accounted for 1.86% of the ASAE dry weight. HPLC analysis detected syringin and eleutheroside E at 0.52 ± 0.19 mg/g and 1.34 ± 0.11 mg/g ASAE dry weight, respectively.

### 3.2 SAE transiently accelerates mycelial biomass accumulation in a concentration- and time-associated manner

**FIGURE 2:**
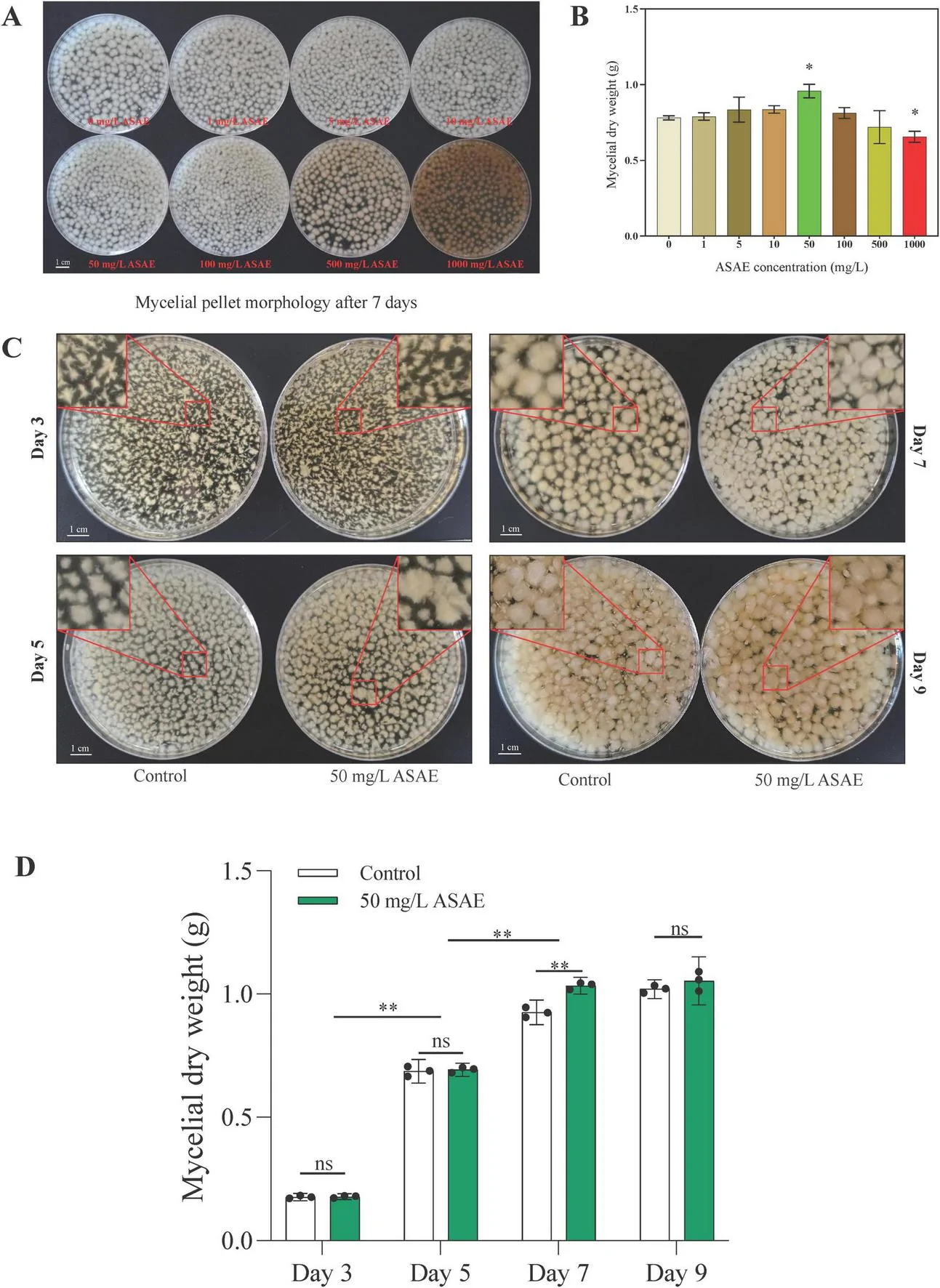
Concentration- and time-associated effects of ASAE on submerged L. decastes mycelial growth. **(A,B)** Representative morphology and mycelial dry weight after treatment with different ASAE concentrations for 7 days. **(C)** Representative images of mycelial aggregates at days 3, 5, 7, and 9. **(D)** Mycelial dry weight during the time-course experiment. Data are presented as mean ± SD from three independent biological replicates. The time-course data were analyzed using two-way ANOVA with cultivation time, treatment, and their interaction as factors, followed by Šidák-adjusted pairwise comparisons. ns, not significant; *p < 0.05; **p < 0.01. Pellet coverage was quantified separately using blinded image analysis of three independent cultivation batches and is reported in the text.

*Acanthopanax senticosus* aqueous extract affected *L. decastes* mycelial growth in a concentration-dependent manner after 7 days of submerged cultivation. Compared with the untreated control, cultures treated with 50 mg/L ASAE formed visibly denser mycelial aggregates and showed the highest dry biomass. The dry weight reached 0.958 ± 0.043 g in the 50 mg/L treatment compared with 0.781 ± 0.014 g in the untreated control, corresponding to a 22.7% increase (*p* < 0.05; Figures 2A,B). In contrast, 1,000 mg/L ASAE significantly reduced dry biomass to 0.655 ± 0.037 g (*p* < 0.05). No visible precipitation or medium turbidity was observed across the tested concentration range. A time-course experiment was subsequently performed using 50 mg/L ASAE. A two-way ANOVA showed significant effects of cultivation time, ASAE treatment, and the time × treatment interaction on mycelial dry weight. Šidák-adjusted comparisons showed no significant difference between Control and ASAE-treated cultures at day 3 or day 5, a significant difference at day 7, and no significant difference at day 9 (Figures 2C,D).

At day 3, both groups showed early mycelial outgrowth and comparable dry weights. At day 5, pellet-like aggregates were visible in both groups, but the difference in dry biomass was not statistically significant. At day 7, ASAE-treated cultures showed visibly denser aggregates and significantly higher dry biomass than the control. By day 9, the dry weights of the two groups were comparable. These findings indicate that 50 mg/L ASAE transiently accelerated biomass accumulation during the mid-to-late cultivation period but did not increase the final biomass yield measured at day 9 under the conditions tested.

Blinded two-dimensional image analysis further supported the visual difference in pellet coverage. Across three independent cultivation batches, pellet coverage was 68%, 70%, and 71% in the control cultures and 75%, 77%, and 76% in the ASAE-treated cultures, respectively. The paired Control-ASAE difference was 6.33 percentage points, with a two-tailed paired *t*-test result of *t* = 9.53, df = 2, and *p* = 0.0108. Because pellet number, size distribution, circularity, and internal structure were not comprehensively quantified, these data are interpreted as evidence of increased visible pellet coverage rather than definitive evidence of altered pellet compactness or internal oxygen-transfer properties. Based on the time-resolved biomass phenotype, days 3, 5, and 7 were selected for transcriptomic analysis to represent early, intermediate, and biomass-accumulating sampling points. These labels indicate sampling times and observed response patterns rather than mechanistically defined developmental stages.

### 3.3 Transcriptome sequencing, *de novo* assembly, and functional annotation

Transcriptome sequencing of 21 *L. decastes* mycelial samples generated 150.02 Gb of clean data, with an average of 7.14 Gb per sample and a minimum of 6.11 Gb per sample. All libraries showed high sequencing quality, with Q30 values ≥ 95.1% and stable GC content of 53.6% ± 0.3% (Supplementary Table 1). Read-to-assembly mapping rates ranged from 92.36% to 95.35% across samples, indicating that the assembled transcriptome captured the majority of sequencing reads.

*De novo* assembly generated 104,960 transcripts and 51,374 unigenes, with a unigene N50 length of 2,139 bp (Supplementary Table 2). Among these assembled sequences, 51,361 unigenes (99.97%) and 104,452 transcripts (99.50%) showed detectable expression in at least one sample. BUSCO assessment of the unigene set showed 100.0% completeness using the fungi_odb10 lineage dataset, including 61.3% complete single-copy and 38.7% complete duplicated BUSCOs, with no fragmented or missing BUSCOs detected (Supplementary Table 2). These results indicated high transcriptome completeness, while the duplicated BUSCOs likely reflected transcript-level redundancy, retained isoforms, allelic variants, closely related paralogs, or transcript variants commonly observed in *de novo* transcriptome assemblies, rather than necessarily indicating genome-level gene duplication.

Functional annotation against public databases showed that 42,550 of 51,374 unigenes (82.82%) had matches in the NR database. Annotation coverage was 64.8% in GO, 40.6% in KEGG, 47.6% in eggNOG, 51.1% in Swiss-Prot, and 54.0% in Pfam, and 12,841 unigenes (25.0% of the total) were annotated in all six databases (Supplementary Table 3). NR-based taxonomic classification showed that most annotated unigenes were assigned to fungal taxa, accounting for 35,140 of 42,550 NR-annotated unigenes (82.6%) (Supplementary Table 4). At the species level, *L. shimeji* and *L. atratum* represented the two largest NR annotation groups, accounting for 32.4% and 20.62% of NR-assigned hits, respectively (Supplementary Figure 3). The predominance of *Lyophyllum*-related matches was consistent with the taxonomic identity of the sequenced mycelial samples and did not suggest substantial contamination by unrelated organisms.

### 3.4 Sample-to-sample expression similarity and principal component analysis

Sample-to-sample correlation analysis showed high overall reproducibility among biological replicates (Supplementary Figure 4 and Supplementary Table 5). The intra-group Pearson correlation coefficients averaged 0.95 ± 0.05 for the untreated control groups (B0, B3, B5, and B7), with values ranging from 0.863 to 0.998, and 0.89 ± 0.05 for the ASAE-treated groups (T3, T5, and T7), with values ranging from 0.818 to 0.953. Although B5 and T3 exhibited slightly lower replicate consistency than B3 and T5, their within-group correlations remained high, with mean Pearson correlation coefficients of 0.901 and 0.863, respectively. The lowest inter-group similarity was observed between B7 and T7, indicating the greatest transcriptional divergence between control and ASAE-treated samples at day 7. Because this stage also showed clear differences in biomass accumulation and pellet morphology, the divergence may reflect both ASAE-responsive transcriptional changes and secondary physiological differences associated with altered growth status.

**FIGURE 3:**
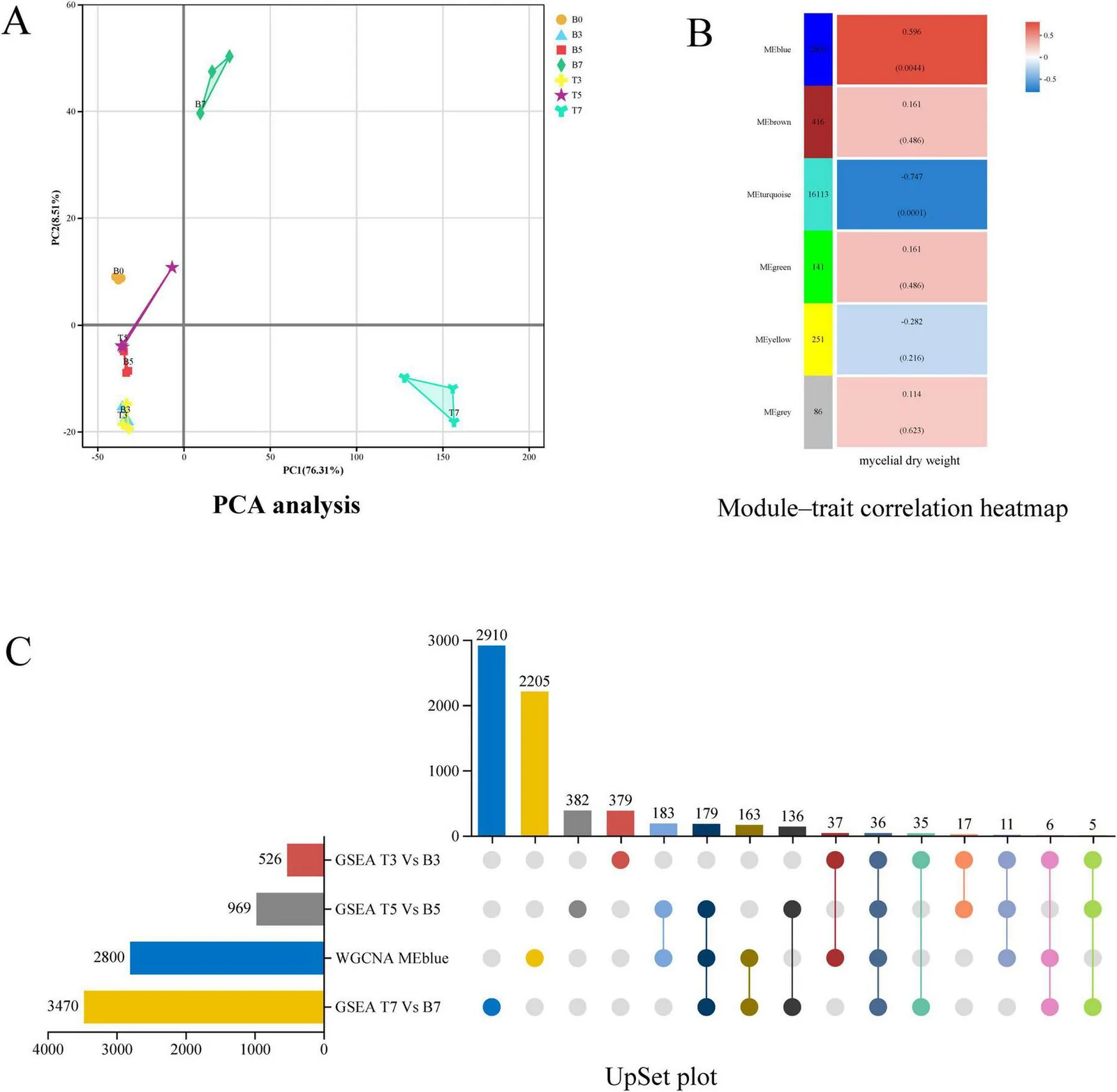
RNA-seq sample relationships and integrative identification of ASAE-responsive biomass-associated gene subsets. **(A)** Principal component analysis of 21 RNA-seq samples based on TPM-normalized unigene expression data. PC1 and PC2 explained 76.31% and 8.51% of the total variance, respectively. **(B)** Module–trait correlation heatmap from WGCNA. Cells show Pearson correlation coefficients between module eigengenes and phenotypic traits, with corresponding *p*-values in parentheses. MEblue showed the strongest positive correlation with mycelial dry weight. **(C)** UpSet plot showing intersections between MEblue genes and GSEA-derived leading-edge genes from the T3 vs. B3, T5 vs. B5, and T7 vs. B7 comparisons. The stage-specific and shared intersected gene sets were used for downstream GO and KEGG enrichment analyses.

Principal component analysis further confirmed clear sample clustering according to cultivation stage and treatment condition (Figure 3A). PC1 explained 76.31% of the total variance and primarily separated samples according to developmental progression and ASAE response: early and intermediate samples, including B0, B3, T3, B5, and T5, clustered closely in the negative PC1 region, whereas B7 and T7 occupied opposite extremes of PC1. PC2 explained 8.51% of the variance and further separated B0 from the later control samples while also distinguishing B7 from T7. Together, the correlation and PCA results confirmed the reproducibility of the RNA-seq dataset and supported its suitability for subsequent WGCNA and GSEA analyses.

### 3.5 Time-resolved transcriptional signatures associated with ASAE treatment

An integrated WGCNA–GSEA framework was used to identify transcriptional signatures associated with ASAE treatment over time. MEblue genes were intersected only with leading-edge genes from positively enriched GSEA gene sets for candidate-gene visualization.

#### 3.5.1 Time and treatment effects on WGCNA module eigengenes

After expression filtering, 19,807 expressed unigenes were retained for WGCNA. Six co-expression modules were identified using β = 16 (Supplementary Figures 5, 6). Among these modules, MEblue contained 2,800 genes and showed the highest module significance for mycelial dry weight, which was used as the biomass trait, with an MS value of 0.7351 (Supplementary Figure 7). Module eigengene–trait correlation analysis further showed that MEblue had the strongest positive correlation with mycelial dry weight, *r* = 0.596, *p* = 0.0044 (Figure 3B). Because both gene expression and biomass changed substantially over cultivation time, module eigengenes were additionally analyzed using linear models containing cultivation time, ASAE treatment, and their interaction.

MEblue showed significant effects of cultivation time (F2,15 = 146.069, adjusted *p* = 1.13 × 10^–8^), treatment (F1,15 = 28.237, adjusted *p* = 2.76 × 10^–4^), and the time × treatment interaction (F2,15 = 17.084, adjusted *p* = 3.08 × 10^–4^). Similar significant interaction effects were observed for MEbrown, MEgreen, MEturquoise, and MEyellow, whereas MEgrey showed no significant time, treatment, or interaction effect after multiple-testing correction (Supplementary Table 6). These results indicate that MEblue captured a co-expression pattern associated with both cultivation progression and ASAE treatment, with the treatment effect varying over time. Therefore, MEblue was interpreted as a time- and treatment-associated module that tracked the biomass phenotype, rather than as a treatment-specific regulatory module. The analysis does not establish that MEblue genes are required for biomass accumulation. By contrast, MEturquoise was negatively correlated with this trait. These results identified MEblue as the co-expression module most positively associated with mycelial dry weight in this dataset and supported its use for downstream integration with GSEA-derived leading-edge gene sets.

#### 3.5.2 Direction-separated GSEA and MEblue-associated candidate subsets

**FIGURE 4:**
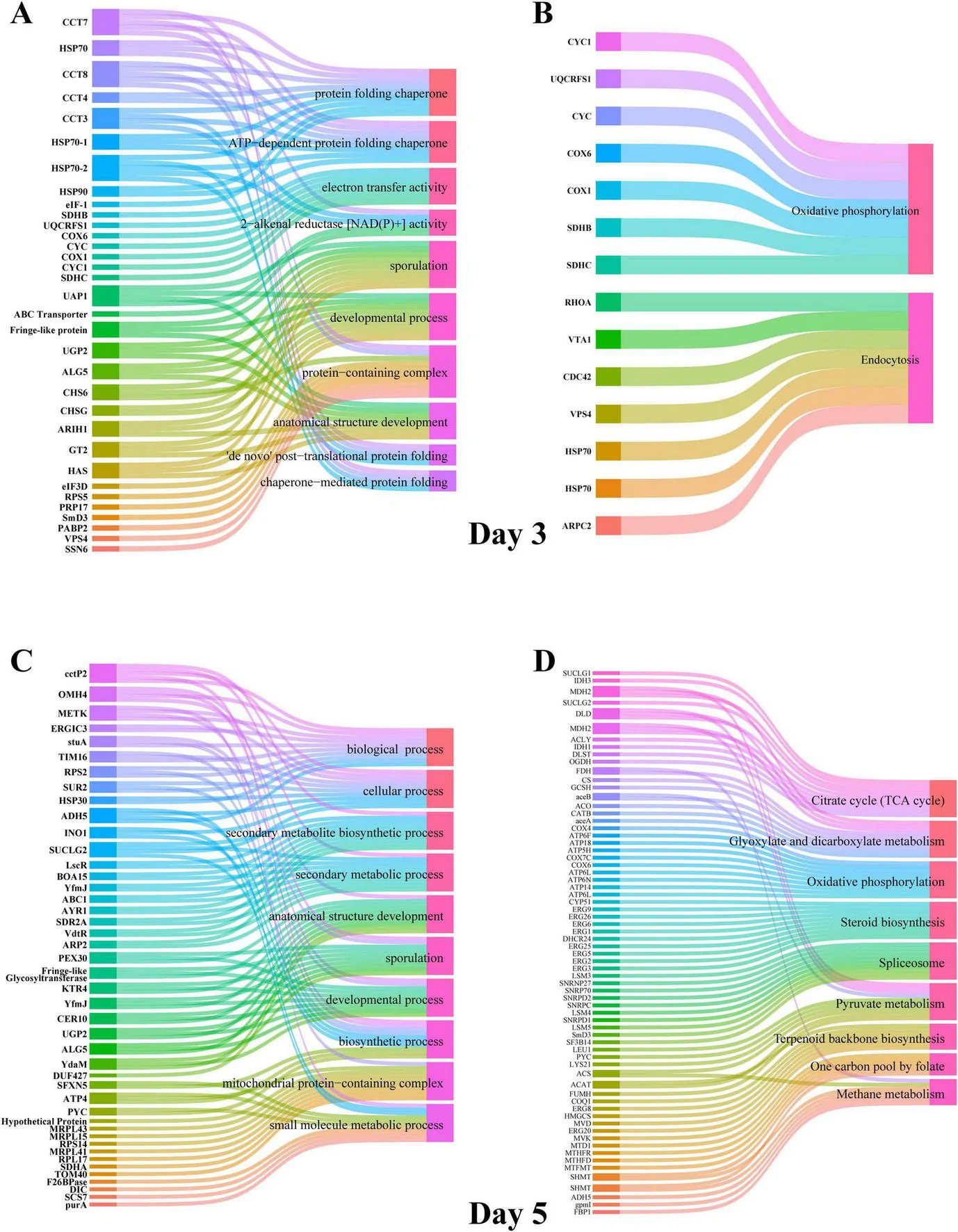
Visualization of direction-separated GSEA results and MEblue-associated positive leading-edge genes at days 3 and 5. **(A,B)** Positive GO- and KEGG-based GSEA gene sets from the T3 versus B3 comparison and their corresponding genes shared with the MEblue module. **(C,D)** Positive GO- and KEGG-based GSEA gene sets from the T5 versus B5 comparison and their corresponding genes shared with the MEblue module. Positive enrichment scores indicate enrichment toward ASAE-treated samples. The displayed gene–term and gene–pathway relationships were obtained by intersecting MEblue genes with leading-edge genes from positively enriched GSEA gene sets at the corresponding sampling time. Functional terms and pathways shown in the figure were derived from the original direction-separated GSEA output. No additional GO or KEGG over-representation analysis was performed on the intersected gene subsets. These visualizations describe time-resolved transcriptional associations and do not imply pathway activation or causal regulation.

**FIGURE 5:**
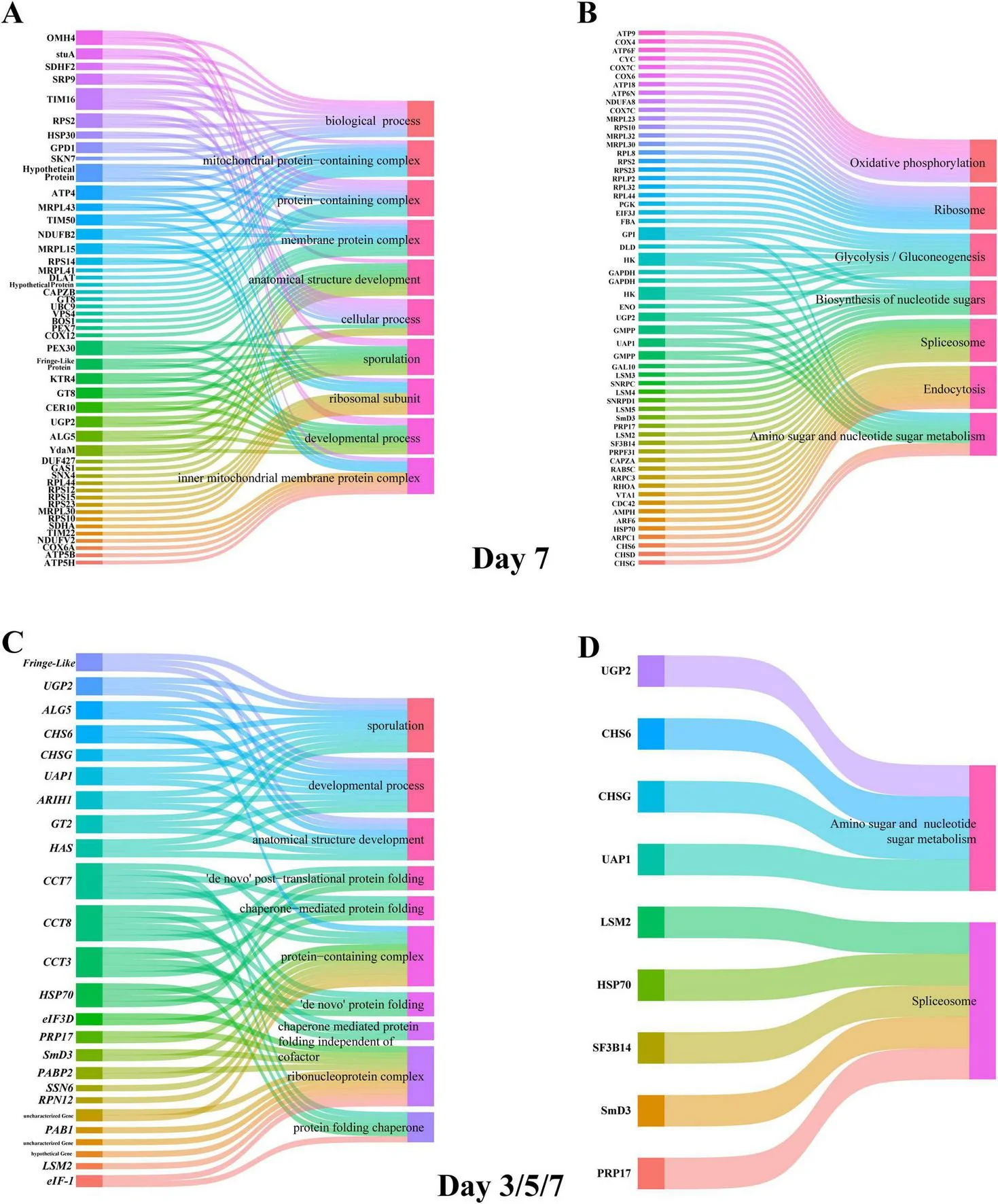
Visualization of direction-separated GSEA results and MEblue-associated positive leading-edge genes at day 7 and across days 3, 5, and 7. **(A,B)** Positive GO- and KEGG-based GSEA gene sets from the T7 versus B7 comparison and their corresponding genes shared with the MEblue module. **(C,D)** GO- and KEGG-based GSEA gene sets represented by genes recurrently shared between MEblue and positively enriched GSEA leading-edge genes at days 3, 5, and 7. Positive enrichment scores indicate enrichment toward ASAE-treated samples. The day-7 and recurrent gene subsets were obtained by intersecting MEblue genes with leading-edge genes from positively enriched GSEA gene sets. The recurrent subset comprised genes retained in these positive MEblue-leading-edge intersections at all three sampling times. Functional terms and pathways shown in the figure were derived from the original direction-separated GSEA output. No additional GO or KEGG over-representation analysis was performed on either the day-7 or recurrent intersected gene subset. Accordingly, recurrence across days should be interpreted as descriptive overlap among ASAE-associated leading-edge genes rather than independent statistical evidence of persistent pathway enrichment, pathway activation, or causal regulation.

GSEA results for the T3 versus B3, T5 versus B5, and T7 versus B7 comparisons are provided in Supplementary Table 7. Positive NES values represented enrichment toward ASAE-treated samples, whereas negative NES values represented enrichment toward untreated controls. MEblue genes were intersected with leading-edge genes from positively enriched gene sets at each sampling time. UpSet analysis summarized the stage-specific and shared MEblue-derived intersections (Figure 3C and Supplementary Table 8). The resulting gene sets were used to visualize the gene–pathway relationships shown in Figures 4, 5. In addition, genes shared across the positive MEblue-leading-edge intersections from days 3, 5, and 7 were defined as a recurrent candidate subset. This shared subset represented genes that were consistently retained in the MEblue module and occurred in positively enriched ASAE-associated leading-edge gene sets at all three sampling times. Because the genes were initially selected as leading-edge members of GO or KEGG gene sets, the intersected subsets were not subjected to a second GO or KEGG enrichment analysis. Accordingly, Figures 4, 5 present the original GSEA enrichment results and the corresponding MEblue-associated leading-edge genes rather than independent over-representation analyses.

At day 3, positively enriched ASAE-associated gene sets included functions related to protein folding, electron transfer, RNA processing, and intracellular trafficking (Figures 4A,B). At day 5, positively enriched gene sets included the citrate cycle, glyoxylate and dicarboxylate metabolism, pyruvate metabolism, oxidative phosphorylation, steroid biosynthesis, and spliceosome-related processes (Figures 4C,D). At day 7, positively enriched gene sets included oxidative phosphorylation, ribosome, glycolysis/gluconeogenesis, nucleotide-sugar biosynthesis, amino sugar and nucleotide sugar metabolism, endocytosis, and spliceosome-related processes (Figures 5A,B). The recurrent day 3/5/7 candidate subset included genes linked in the original GSEA output to amino sugar and nucleotide sugar metabolism, spliceosome-related functions, protein folding, and chaperone-mediated processes (Figures 5C,D). Thus, these functional categories were repeatedly represented among the positively enriched, MEblue-associated leading-edge genes across the three sampling times. This recurrence is consistent with persistent transcriptional association of ASAE treatment with RNA-processing, protein-homeostasis, and carbohydrate-metabolism-related functions. However, because the shared subset was derived from the original GSEA leading-edge genes, these observations should be interpreted as descriptive recurrence across time rather than as independent statistical evidence of persistent pathway enrichment or functional activation.

#### 3.5.3 Sensitivity analysis of transcript redundancy

The *de novo* assembly contained 51,374 unigenes and 104,960 transcripts. BUSCO analysis identified duplicated proportions of 38.7% for the unigene set and 46.1% for the transcript set. To examine whether transcript redundancy influenced the main conclusions, expressed unigenes were collapsed into orthologue-level units using eggNOG orthogroup, KO, and Swiss-Prot annotations. When multiple unigenes were assigned to the same unit, the unigene with the highest mean expression across all samples was retained as the representative transcript.

The collapsed expression matrix was reanalyzed using the same WGCNA and direction-separated GSEA workflow. The main results were qualitatively consistent with the original unigene-level analysis (Supplementary Table 9). MEblue remained significantly associated with cultivation time, ASAE treatment, and their interaction. Major time-resolved enrichment patterns involving energy metabolism, RNA processing, intracellular trafficking, translation, and carbohydrate-associated metabolism were retained. *CHSG*, *UAP1*, *ALG5*, and *RPN12* were also represented in the collapsed analysis. This sensitivity analysis supports the qualitative robustness of the main transcript-level associations. However, because no species-specific reference genome was available and homology-based collapse cannot fully resolve paralogs, isoforms, and allelic variants, the results remain transcript-level and hypothesis-generating.

### 3.6 RT-qPCR validation supports the RNA-seq-derived expression trends

Seven candidate reference genes, including α-tubulin, β-actin, *GAPDH*, ATP citrate lyase (*ACLY*), 60S ribosomal protein L3 (*RPL3*), 60S ribosomal protein L7 (*RPL7*), and V-type H^+-transporting ATPase 16 kDa proteolipid subunit (*V-ATP*), were evaluated for RT-qPCR normalization across all samples. RefFinder-based comprehensive ranking identified α-tubulin as the most stable candidate, followed by GAPDH and β-actin, whereas *V-ATP* showed the lowest stability (Supplementary Figure 8A). The comparative ΔCt and NormFinder analyses also ranked α-tubulin, *GAPDH*, and β-actin among the most stable candidates, and geNorm identified α-tubulin and *GAPDH* as the optimal reference gene pair, with an *M*-value of 0.504 (Supplementary Figures 8B–D). Therefore, the geometric mean of α-tubulin and *GAPDH* expression levels was used for normalization in subsequent RT-qPCR analyses.

To validate the RNA-seq-derived expression trends, 13 representative ASAE-responsive genes were selected for RT-qPCR analysis. These genes were associated with major functional categories identified by the WGCNA–GSEA analysis, including protein synthesis, energy metabolism, cell-wall biosynthesis, glycosylation, membrane trafficking, and proteostasis. The RT-qPCR experiments used newly prepared biological samples and therefore constituted independent biological confirmation rather than technical remeasurement of the RNA-seq libraries. The selected genes were analyzed either at their corresponding sampling stages or, for selected candidates, across multiple time points, yielding 21 gene–time point combinations across days 3, 5, and 7. Four genes, *CHSG*, *ALG5*, *UAP1*, and *RPN12*, were further examined across all three time points to assess temporal expression patterns. Primer amplification efficiencies ranged from 98.11% to 102.85%, with R^2^ values of 0.997–0.999, indicating reliable amplification performance (Supplementary Table 10). The Ct values used for RT-qPCR-based relative expression analysis are also provided in Supplementary Table 10.

**FIGURE 6:**
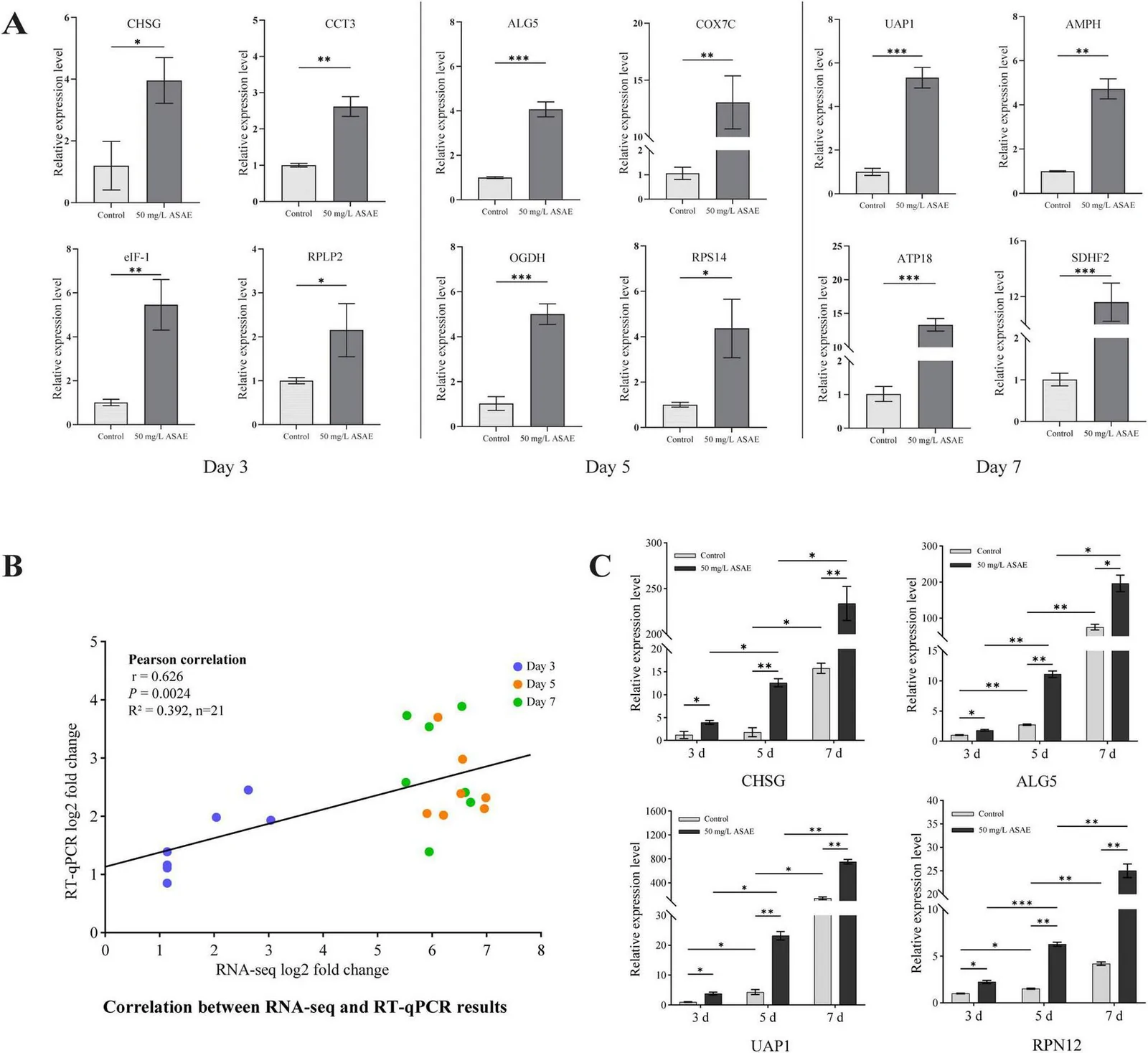
RT-qPCR validation of RNA-seq-derived expression trends and temporal expression of selected ASAE-responsive genes. **(A)** RT-qPCR validation of selected ASAE-responsive candidate genes at their corresponding sampling stages. Expression levels were normalized using the geometric mean of α-tubulin and GAPDH and calculated using the 2^−ΔΔCt method, with the corresponding untreated control at each time point as the calibrator. **(B)** Correlation between RNA-seq- and RT-qPCR-derived log2 fold-change values across 21 gene–time point combinations. Pearson correlation analysis was used to assess concordance between the two datasets. **(C)** Temporal expression patterns of *CHSG*, *ALG5*, *UAP1*, and *RPN12* in control and ASAE-treated samples across days 3, 5, and 7. The day 3 control was used as the baseline calibrator. Data are presented as mean ± SD, *n* = 3 biological replicates. Statistical significance was assessed as described in the Materials and Methods. *P < 0.05; **p < 0.01; ***p < 0.001.

RT-qPCR analysis of selected genes at their corresponding sampling stages confirmed the ASAE-associated higher expression patterns observed in the RNA-seq dataset (Figure 6A). All 21 gene–time point combinations showed higher expression in ASAE-treated samples than in their corresponding same-time-point controls, consistent with the RNA-seq results (Supplementary Table 10). The RT-qPCR-derived fold changes ranged from 1.80 to 14.84, whereas the RNA-seq-derived fold changes ranged from 2.21 to 126.93. Pearson correlation analysis further showed a significant positive correlation between RNA-seq- and RT-qPCR-derived log2 fold-change values across the 21 validation points (Pearson’s *r* = 0.626, *p* = 0.0024; Figure 6B), supporting the reliability of the transcriptome-derived expression trends.

The temporal expression profiles of *CHSG*, *ALG5*, *UAP1*, and *RPN12* were then examined across days 3, 5, and 7 using the day 3 control as the baseline calibrator (Figure 6C). In the control group, CHSG expression did not differ markedly between days 3 and 5 but increased at day 7, whereas *ALG5*, *UAP1*, and *RPN12* showed progressive increases over time. Under ASAE treatment, all four genes showed higher expression than their corresponding controls at each time point and also displayed increasing expression over the cultivation period. These results indicate that ASAE treatment was associated with sustained upregulation of selected candidate genes related to cell-wall-associated carbohydrate metabolism, glycosylation, UDP-N-acetylglucosamine metabolism, and proteasome-associated protein turnover.

## 4 Discussion

### 4.1 ASAE transiently accelerates *L. decastes* mycelial biomass accumulation in a concentration- and time-associated manne

Our results show that ASAE was associated with a transient acceleration of *L. decastes* mycelial biomass accumulation during submerged cultivation, and that the response depended on both ASAE concentration and cultivation time. Among the tested concentrations, 50 mg/L ASAE produced the greatest increase in mycelial dry weight at day 7, whereas 1,000 mg/L significantly suppressed growth. In the time-course experiment, two-way ANOVA identified significant effects of cultivation time, ASAE treatment, and the time × treatment interaction on mycelial dry weight. Subsequent adjusted pairwise comparisons showed a significant treatment-associated difference at day 7, but not at days 3, 5, or 9. Thus, under the conditions tested, ASAE transiently accelerated biomass accumulation around day 7 rather than increasing the final biomass yield measured at day 9.

The time-course observations further showed that ASAE did not markedly affect early mycelial establishment. Instead, the treatment-associated biomass difference became apparent during the mid-to-late cultivation period, when ASAE-treated cultures showed visibly denser mycelial aggregates and higher visible pellet coverage. These observations suggest that ASAE acts within a limited effective concentration range and is associated with altered macroscopic mycelial morphology and transient biomass accumulation rather than with the initial establishment of mycelial growth. However, pellet number, size distribution, circularity, density, internal structure, and oxygen-transfer properties were not comprehensively measured. Therefore, the observed morphological differences should not be interpreted as direct evidence of pellet compaction or its physiological consequences.

The growth-associated response should be interpreted in the context of the chemical complexity of ASAE. ASAE is a hot-water plant extract rather than a single purified compound. In this study, the phenol–sulfuric acid assay indicated that total carbohydrate-equivalent content accounted for 1.86% of the ASAE dry weight. On this basis, the 50 mg/L treatment introduced approximately 0.93 mg/L carbohydrate equivalents into the PDB medium. This amount is low relative to the carbon already present in PDB, making it unlikely that the observed day-7 biomass difference was caused solely by the measured carbohydrate-equivalent fraction. However, the phenol–sulfuric acid assay is not specific for structurally defined polysaccharides in an unfractionated extract and may also detect free sugars and oligosaccharides. In addition, ASAE may contain a mixture of water-soluble constituents, including carbohydrates, phenolics, lignans, nitrogen-containing compounds, minerals, vitamins, organic acids, and other small molecules, which may contribute through nutritional, physicochemical, elicitor-like, or stress-adaptive effects (Davydov and Krikorian, 2000; Huang et al., 2011; Jia et al., 2021). Although syringin and eleutheroside E were detected as characteristic constituents, the present study cannot assign the observed response to individual compounds. Therefore, the ASAE effect should be considered the combined effect of a partially characterized plant-derived aqueous extract.

The biphasic concentration response, in which 50 mg/L ASAE was associated with higher biomass at day 7 whereas 1,000 mg/L inhibited growth, may be compatible with a hormesis-like response. Low concentrations of chemically complex plant extracts may potentially act as mild stress cues or elicitor-like stimuli, while higher concentrations may exert inhibitory nutritional, chemical, osmotic, or other physicochemical effects. Consistent with this possibility, positively enriched gene sets at day 3 included protein-folding and chaperone-associated functions, suggesting that proteostasis-related transcriptional responses may accompany early cellular adjustment. However, the present data do not directly demonstrate ER stress, unfolded protein response activation, ROS-mediated signaling, or hormesis. Moreover, matched carbon, nitrogen, mineral, pH, and osmotic controls were not included. Therefore, the nutritional, elicitor-like, and stress-adaptive interpretations remain hypothetical and require further validation.

### 4.2 ASAE-associated transient biomass accumulation is accompanied by time-resolved transcriptional patterns

The WGCNA–GSEA analysis indicates that ASAE-associated transient biomass accumulation was accompanied by time-resolved transcriptional patterns. However, these data do not demonstrate that MEblue genes are functionally required for the observed phenotype. In the original module–trait analysis, MEblue showed the strongest positive correlation with mycelial dry weight. Because both biomass and global gene expression changed substantially with cultivation time, module eigengenes were further analyzed using models including cultivation time, ASAE treatment, and their interaction. MEblue showed significant effects of time, treatment, and time × treatment interaction. Thus, MEblue is interpreted here as a time- and treatment-associated co-expression module that tracks the biomass phenotype, rather than as an ASAE-specific biomass-regulatory module.

At day 3, positively enriched ASAE-associated gene sets included functions related to oxidative phosphorylation, endocytosis, spliceosome activity, and protein folding. Because no significant treatment-associated biomass difference was detected at day 3, these patterns should not be interpreted as direct evidence of biomass production. Instead, they may reflect transcriptional differences related to energy metabolism, vesicle trafficking, RNA processing, and protein homeostasis during early cultivation. Such functions are biologically relevant to filamentous fungal growth because polarized hyphal extension involves ATP demand, endocytic recycling, vesicle-mediated delivery of membrane and wall materials, and protein quality control (Steinberg, 2007; Riquelme et al., 2011; Hartl et al., 2011; Balchin et al., 2016). Several genes annotated as protein kinases, calcium-binding proteins, or oxidoreductases were detected among the day-3 ASAE-associated candidates, but canonical MAPK-, calcium-, or ROS-signaling pathways were not significantly enriched. Accordingly, the present data do not demonstrate activation of these signaling pathways.

At day 5, the positive enrichment profile included pathways related to the citrate cycle, glyoxylate and dicarboxylate metabolism, pyruvate metabolism, oxidative phosphorylation, steroid biosynthesis, and spliceosome function. This pattern is consistent with time-associated transcriptional differences in central carbon metabolism, mitochondrial functions, membrane-related biosynthetic processes, and RNA processing before the significant day-7 biomass difference became apparent. Higher transcript abundance of genes assigned to carbon oxidation- and precursor-related pathways may be compatible with altered metabolic requirements during cultivation, while steroid biosynthesis-related transcriptional changes may be associated with membrane remodeling. However, these data do not establish increased carbon utilization, altered carbon flux, increased respiration, increased sterol synthesis, or metabolic support for subsequent biosynthesis.

The enrichment of glyoxylate and dicarboxylate metabolism at day 5 was notable because PDB is a glucose-rich medium. This response may reflect local rather than bulk nutrient conditions, differences in culture morphology, developmental state, or other cultivation-associated variables. As mycelial aggregates enlarge, gradients of glucose, oxygen, and metabolic by-products may develop within aggregate interiors, potentially contributing to central carbon metabolic remodeling. Alternatively, ASAE may introduce minor carbon sources or other water-soluble constituents that influence transcriptional responses related to carbon metabolism. Because residual glucose, dissolved oxygen, intracellular organic acids, aggregate microenvironments, and carbon flux were not measured in the present study, these possibilities remain speculative.

The day-7 transcriptional response coincided with the transient treatment-associated increase in dry biomass and higher visible pellet coverage. Positively enriched gene sets at this time point included oxidative phosphorylation, ribosome, glycolysis/gluconeogenesis, nucleotide-sugar biosynthesis, amino sugar and nucleotide sugar metabolism, endocytosis, and spliceosome-related pathways. These associations are biologically compatible with transcriptional remodeling in functional categories related to energy metabolism, translation, carbohydrate metabolism, intracellular trafficking, and cell-wall-associated biosynthetic processes. In particular, amino sugar and nucleotide sugar metabolism and nucleotide-sugar biosynthesis are relevant to fungal cell-wall construction because these pathways include activated sugar donors used in chitin, glucan, and glycoprotein-associated carbohydrate biosynthesis (Free, 2013; Hopke et al., 2018). Nevertheless, the present transcriptomic data do not directly demonstrate increased ATP production, enhanced respiration, higher carbon flux, greater UDP-GlcNAc availability, increased chitin or glucan synthesis, increased endocytosis, or increased cell-wall precursor supply.

The pronounced B7-T7 transcriptional divergence may also reflect secondary physiological differences associated with transient biomass accumulation, altered macroscopic aggregate morphology, nutrient utilization, oxygen availability, or developmental state. Therefore, the day-7 comparison should not be interpreted as direct ASAE regulation alone. Overall, the findings support a tentative time-associated hypothesis in which ASAE treatment is accompanied by early transcriptional signatures related to cellular adjustment, followed by signatures related to central metabolism and, at day 7, by signatures associated with biosynthetic and structural functional categories during transient biomass accumulation. This hypothesis requires direct physiological and functional testing.

### 4.3 Candidate genes are associated with cell-wall-related carbohydrate metabolism, glycosylation, and proteostasis

Independent RT-qPCR experiments supported the RNA-seq-derived expression trends of the selected candidate genes. Across 21 gene–time point combinations, all selected genes showed higher expression in ASAE-treated samples than in their corresponding controls, and RNA-seq- and RT-qPCR-derived log2 fold-change values were significantly correlated. Although the absolute fold-change values differed between the two methods, such differences are common because RNA-seq and RT-qPCR differ in dynamic range, normalization strategy, transcript quantification, and primer-specific amplification. Thus, the RT-qPCR results provide independent biological confirmation of the expression direction and temporal trends of the selected transcripts.

However, the selected genes were derived from the RNA-seq candidate set. Therefore, RT-qPCR does not independently validate WGCNA module function, GSEA pathway activity, or causal involvement in biomass accumulation. Instead, it confirms that selected transcript-level differences were reproducible in independent biological experiments.

Among the validated candidates, *CHSG*, *UAP1*, *ALG5*, and *RPN12* have annotations relevant to the time-associated working hypothesis. CHSG encodes a chitin synthase associated with chitin biosynthesis, a key process in fungal cell-wall construction and remodeling (Free, 2013). *UAP1* is involved in UDP-N-acetylglucosamine metabolism, which is associated with activated amino sugar metabolism relevant to chitin and other glycoconjugates (Milewski et al., 2006; Free, 2013). *ALG5* participates in dolichol-linked glucose donor formation during protein N-glycosylation, a process important for glycoprotein maturation and the function of membrane- or cell-wall-associated proteins (Helenius and Aebi, 2004; Lehle et al., 2006). *RPN12*, a component of the 26S proteasome regulatory particle, is associated with ubiquitin–proteasome-mediated protein turnover and proteome maintenance (Finley et al., 2012).

The sustained ASAE-associated higher expression of these genes is consistent with transcriptional changes in functional categories related to cell-wall-associated carbohydrate metabolism, glycosylation, and protein homeostasis. However, transcript abundance alone does not demonstrate increased chitin synthesis, increased UDP-N-acetylglucosamine availability, enhanced glycoprotein maturation, or higher proteasome activity. Thus, these candidates provide biologically plausible targets for future functional analyses rather than direct evidence of the corresponding biochemical activities.

### 4.4 Working hypothesis and limitations

**FIGURE 7:**
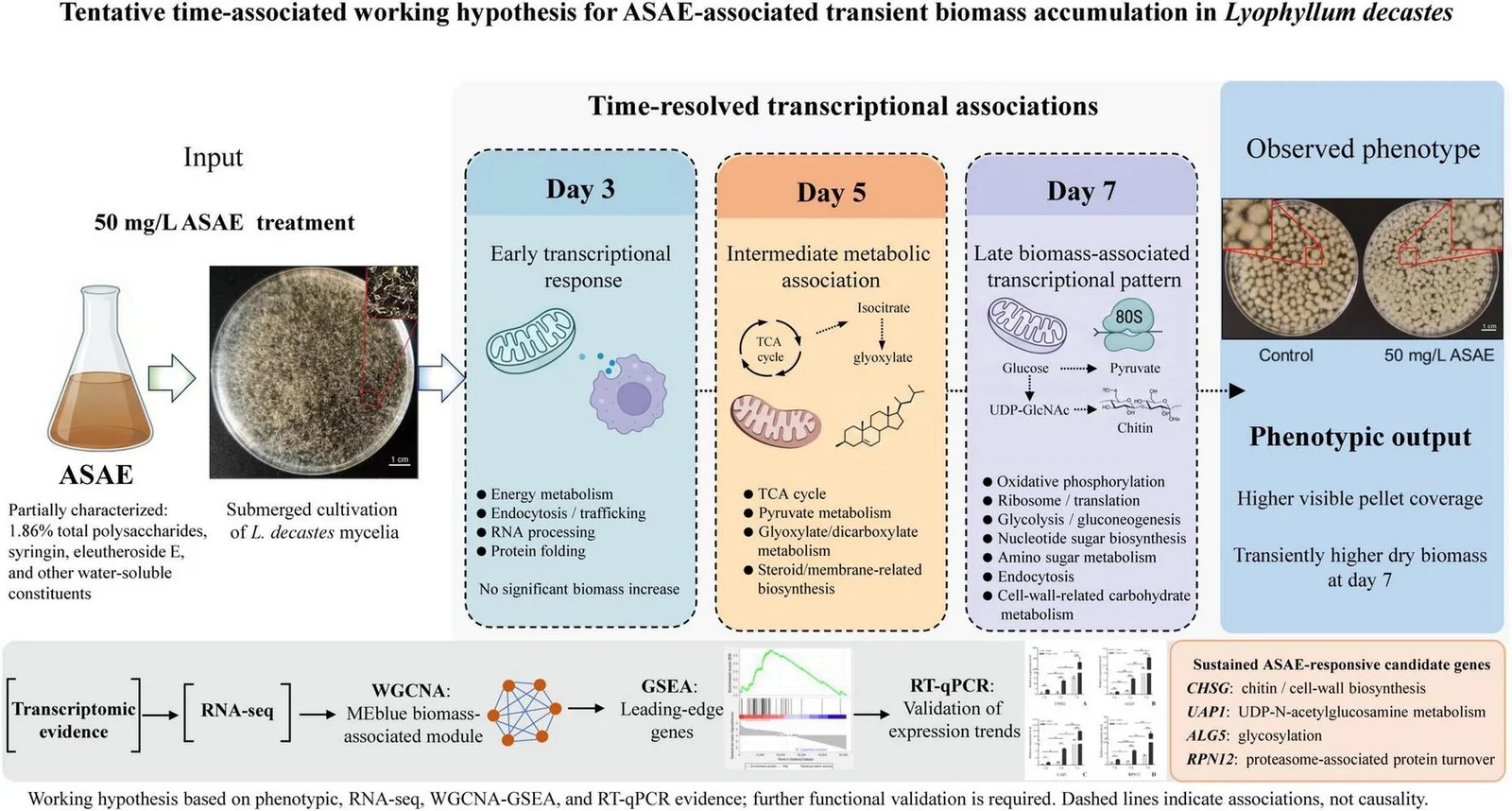
Tentative time-associated working hypothesis for ASAE-associated transient mycelial biomass accumulation in the cultivated *Lyophyllum* isolate provisionally assigned to *L. decastes*. The diagram summarizes phenotypic observations and time-resolved transcriptomic associations observed during submerged cultivation with 50 mg/L ASAE. At day 3, ASAE-associated transcriptional signatures included gene sets related to energy metabolism, intracellular trafficking, RNA processing, and protein homeostasis. At day 5, the associated signatures included central carbon metabolism, mitochondrial-related processes, and membrane-associated biosynthetic functions. At day 7, when a transient increase in mycelial dry weight and higher visible pellet coverage were observed, ASAE-associated transcriptional signatures included oxidative phosphorylation, translation, carbohydrate-associated metabolism, nucleotide-sugar biosynthesis, endocytosis, and cell-wall-related functional annotations. Independent RT-qPCR experiments confirmed the expression trends of selected candidate genes, including *CHSG*, *UAP1*, *ALG5*, and *RPN12*. Dashed lines indicate inferred associations and do not imply direct regulation or causality. The diagram is hypothesis-generating and does not demonstrate increased ATP production, carbon flux, nucleotide-sugar availability, cell-wall synthesis, proteasome activity, or a causal relationship between transcriptomic changes, pellet morphology, and biomass accumulation. Further physiological, biochemical, and functional experiments are required.

Based on the phenotypic, transcriptomic, transcript-collapse sensitivity, and independent RT-qPCR evidence, we propose a tentative time-associated working hypothesis for ASAE-associated transient mycelial biomass accumulation in the cultivated *Lyophyllum* isolate provisionally assigned to *L. decastes* (Figure 7). This hypothesis should be considered exploratory and requires functional validation. In the figure, dashed lines indicate inferred associations rather than demonstrated causal relationships. ASAE is a complex plant-derived aqueous extract that may provide nutritional inputs, physicochemical influences, and/or bioactive cues. Rather than directly accelerating early mycelial initiation, ASAE treatment was associated with early transcriptional signatures related to energy metabolism, intracellular trafficking, RNA processing, and protein homeostasis. This was followed by time-associated transcriptional patterns involving central carbon metabolism and mitochondrial functions. At day 7, when the transient dry-biomass difference was observed, ASAE-associated transcriptional changes included functional categories related to energy metabolism, translation, carbohydrate metabolism, nucleotide-sugar biosynthesis, endocytosis, and cell-wall-associated carbohydrate processes. These observations provide a framework for testable hypotheses but do not establish a molecular mechanism for biomass accumulation or altered aggregate morphology.

Several limitations should be noted. First, ASAE was only partially chemically characterized. The phenol–sulfuric acid assay measured total carbohydrate-equivalent content rather than structurally defined polysaccharides, and the active constituents responsible for the observed response remain unknown. ASAE may also contain free sugars, oligosaccharides, nitrogen-containing compounds, minerals, vitamins, organic acids, phenolics, lignans, and other water-soluble constituents. Fractionation of ASAE, characterization of defined fractions, testing of individual compounds, and matched carbon, nitrogen, mineral, pH, and osmotic controls will be required to distinguish nutritional effects from specific bioactive, elicitor-like, or stress-adaptive effects.

Second, the transcriptomic evidence is association-based. WGCNA and GSEA identify co-expression and gene-set-level patterns but do not directly demonstrate regulatory causality. The present data therefore do not prove increased ATP production, altered carbon flux, enhanced respiration, increased cell-wall polysaccharide synthesis, increased UDP-N-acetylglucosamine availability, increased proteasome activity, or functional involvement of individual genes.

Third, the proposed working hypothesis should be tested using physiological, biochemical, metabolomic, proteomic, and genetic approaches. Future studies should include ATP and respiratory activity assays, residual substrate and dissolved-oxygen measurements, targeted carbon and amino-sugar metabolite profiling, chitin and glucan quantification, sterol profiling, proteasome activity assays, quantitative pellet morphology and internal-structure analyses, Western blotting and/or proteomic analyses, and functional validation of candidate genes such as *CHSG*, *UAP1*, *ALG5*, and *RPN12* using RNAi, CRISPR-based approaches, or overexpression systems.

Fourth, although *de novo* transcriptome assembly was appropriate in the absence of a fully annotated reference genome, transcript redundancy remains a limitation. The homology-based transcript-collapse sensitivity analysis showed that the major module-level and direction-separated GSEA patterns were qualitatively retained after collapsing redundant unigenes into orthologue-level units. However, this approach cannot fully distinguish isoforms, allelic variants, paralogs, or incomplete transcript fragments. Future mapping to a high-quality annotated reference genome for *L. decastes*, together with multilocus or genome-based taxonomic confirmation of LD-DZ2025, would reduce transcript redundancy, improve gene-level quantification, strengthen species assignment, and facilitate functional discovery.

## Data Availability

The original contributions presented in this study are included in this article/Supplementary material, further inquiries can be directed to the corresponding authors.

## Supplementary material

Supplementary Figure 1 Forward ITS sequencing chromatogram of isolate LD-DZ2025.

Supplementary Figure 2 Reverse ITS sequencing chromatogram of isolate LD-DZ2025.

Supplementary Figure 3 Species distribution of NR annotation hits for the *L. decastes* transcriptome. The pie chart shows the species-level distribution of top NR hits among NR-annotated unigenes.

Supplementary Figure 4 Sample-to-sample Pearson correlation heatmap of RNA-seq expression profiles. Pairwise correlations were calculated using TPM-normalized unigene expression data from 21 RNA-seq samples.

Supplementary Figure 5 Selection of the soft-thresholding power for WGCNA. Scale-free topology fit index and mean connectivity were evaluated across candidate powers. A soft-thresholding power of β = 16 was selected for network construction.

Supplementary Figure 6 Gene clustering dendrogram and module assignment by WGCNA. Co-expression modules were identified using topological overlap-based dissimilarity and dynamic tree cutting. Colors indicate assigned modules, and gray indicates genes not assigned to a specific module.

Supplementary Figure 7 Module significance analysis for mycelial dry weight. Module significance was calculated as the mean gene significance of genes within each WGCNA module. MEblue showed the highest module significance for mycelial dry weight.

Supplementary Figure 8 Stability evaluation of candidate reference genes for RT-qPCR normalization. Seven candidate reference genes were evaluated using RefFinder, comparative ΔCt, NormFinder, and geNorm analyses. α-tubulin and GAPDH were selected for normalization of target gene expression.

Supplementary Table 1 Sequencing and quality-control statistics of 21 *L. decastes* RNA-seq libraries. Raw reads, clean reads, Q30 percentage, GC content, and read-to-assembly mapping rate are shown for each sample.

Supplementary Table 2 *De novo* transcriptome assembly statistics and BUSCO assessment. Assembly metrics and BUSCO completeness based on the fungi_odb10 lineage dataset are shown.

Supplementary Table 3 Functional annotation summary of assembled unigenes. Numbers and percentages of unigenes annotated in GO, KEGG, eggNOG, NR, Swiss-Prot, and Pfam databases are shown.

Supplementary Table 4 NR-based taxonomic distribution of annotated unigenes. Taxonomic assignments of NR-annotated unigenes are summarized at higher taxonomic and species levels.

Supplementary Table 5 Sample-to-sample Pearson correlation matrix for RNA-seq samples. Pairwise Pearson correlation coefficients were calculated using TPM-normalized expression data.

Supplementary Table 6 Statistical analysis of WGCNA module eigengenes using models incorporating cultivation time, ASAE treatment, and their interaction.

Supplementary Table 7 Complete GSEA results for ASAE-treated versus control comparisons. Results are shown for T3 vs. B3, T5 vs. B5, and T7 vs. B7, including gene set size, NES, nominal *p*-value, FDR *q*-value, and enrichment direction.

Supplementary Table 8 Functional annotation of MEblue–GSEA intersected gene sets. Separate sheets provide annotations for the MEblue–day 3, MEblue–day 5, MEblue–day 7, and shared MEblue–day 3/5/7 intersected gene sets.

Supplementary Table 9 Results of the homology-based transcript-collapse sensitivity analysis.

Supplementary Table 10 RT-qPCR reference gene evaluation, primer information, Ct values, and validation results. Separate sheets provide candidate reference gene information, reference gene Ct values, target gene primer information, target gene Ct values, and RNA-seq versus RT-qPCR expression comparisons.
