# Supplementary material for: Transient acceleration of Lyophyllum decastes mycelial biomass accumulation by Acanthopanax senticosus extract
Source: Front Microbiol. 2026 Sep 3;17:1894250. doi: 10.3389/fmicb.2026.1894250 (PMC13582560; doi:10.3389/fmicb.2026.1894250)

ITS sequencing chromatogram showing the high-quality forward read for LD-DZ2025.ab1 Signal G:1529 A:4284 T:8092 C:5344
Sample: 71602941896_31623032100159_(L-3)_[M13-26]_PW
756 bases in 9160 scans

Run Ended: Mar 22, 2026, 20:26:26
Comment:
Lane: 24 Base spacing 14.47

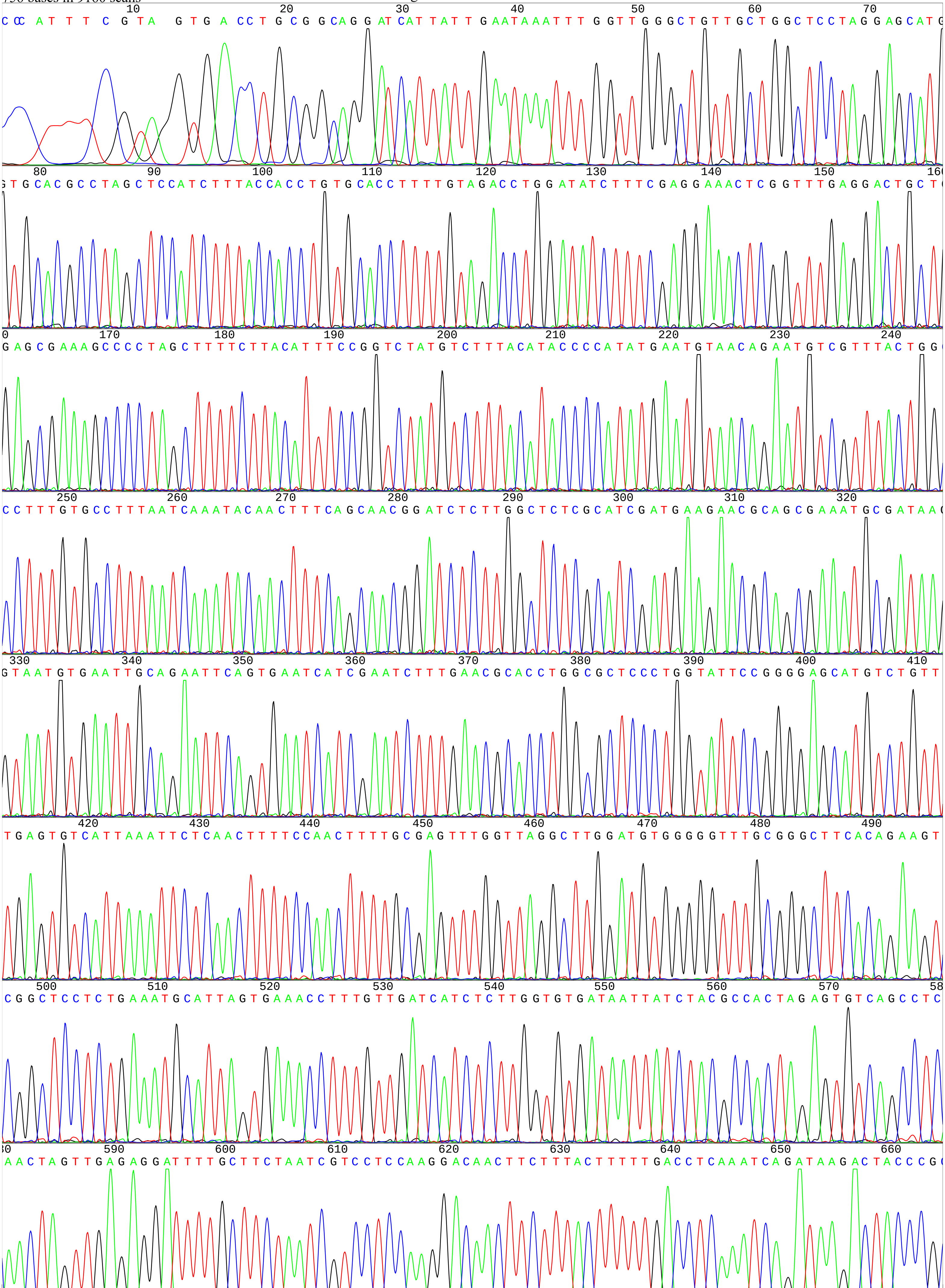

ITS sequencing chromatogram showing the high-quality forward read for LD-DZ2025.ab1 Signal G:1529 A:4284 T:8092 C:5344
Sample: 71602941896_31623032100159_(L-3)_[M13-26]_PW
756 bases in 9160 scans

Run Ended: Mar 22, 2026, 20:26:26
Comment:
Lane: 24 Base spacing 14.47

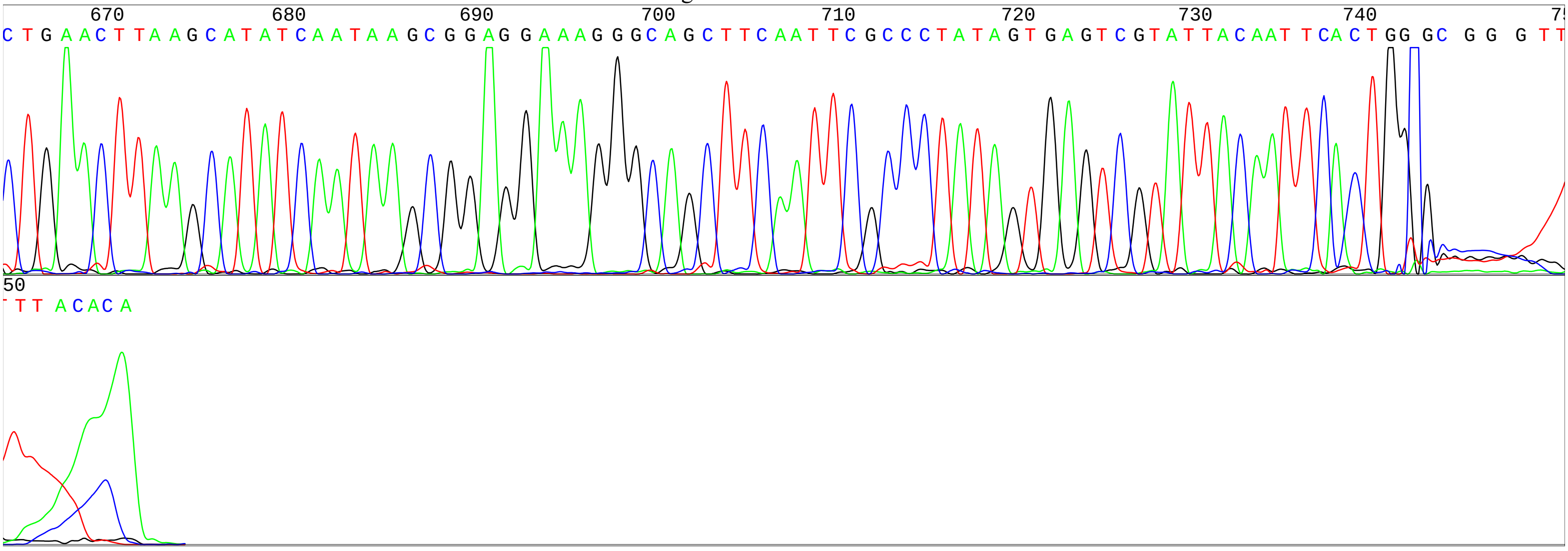

Supplement: Supplementary file 1 [file Data_Sheet_1.pdf]
