# Supplementary material for: Transient acceleration of Lyophyllum decastes mycelial biomass accumulation by Acanthopanax senticosus extract
Source: Front Microbiol. 2026 Sep 3;17:1894250. doi: 10.3389/fmicb.2026.1894250 (PMC13582560; doi:10.3389/fmicb.2026.1894250)

ITS sequencing chromatogram showing the high-quality reverse read for LD-DZ2025 .ab1 Signal G:1189 A:3744 T:5184 C:3914
Sample: 71602941897_31623032100159_(L-3)_[M13-F]_PW
761 bases in 9179 scans

Run Ended: Mar 22, 2026, 20:26:26
Comment:
Lane: 22 Base spacing 14.33

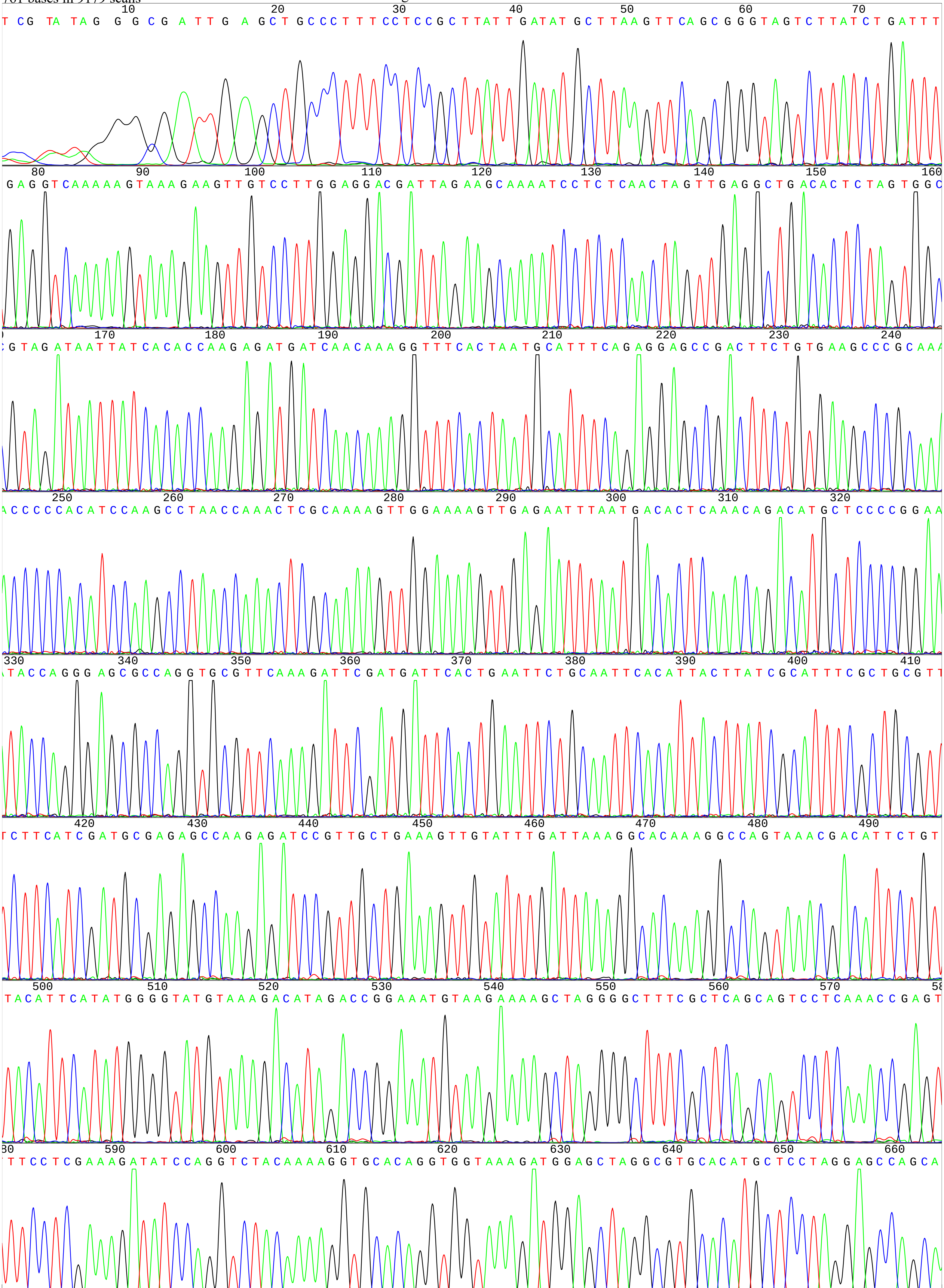

ITS sequencing chromatogram showing the high-quality reverse read for LD-DZ2025.ab1 Signal G:1189 A:3744 T:5184 C:3914
Sample: 71602941897_31623032100159_(L-3)_[M13-F]_PW
761 bases in 9179 scans

Run Ended: Mar 22, 2026, 20:26:26
Comment:
Lane: 22 Base spacing 14.33

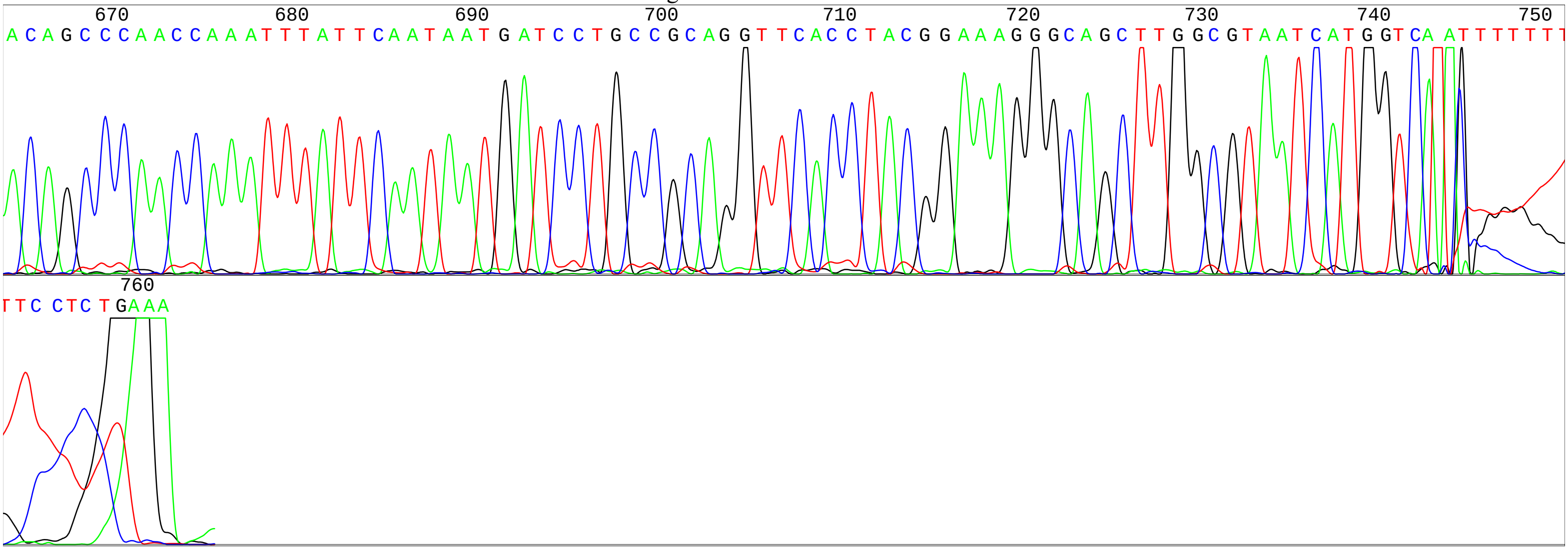

Supplement: Supplementary file 2 [file Data_Sheet_2.pdf]
