# Supplementary material for: Transient acceleration of Lyophyllum decastes mycelial biomass accumulation by Acanthopanax senticosus extract
Source: Front Microbiol. 2026 Sep 3;17:1894250. doi: 10.3389/fmicb.2026.1894250 (PMC13582560; doi:10.3389/fmicb.2026.1894250)
Supplement: Supplementary file 4 [file Data_Sheet_4.pdf]

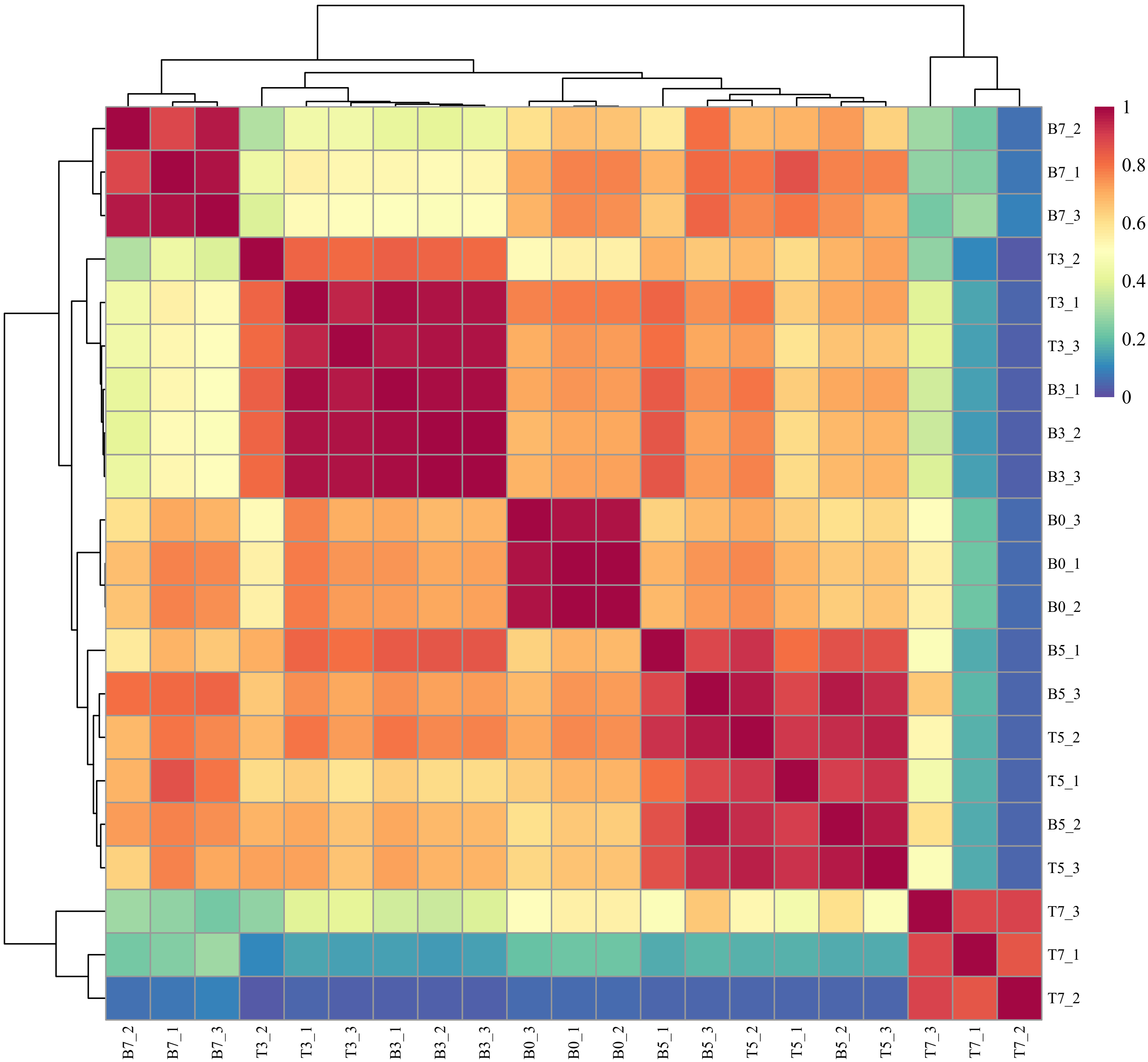

B7_2
B7_1
B7_3
T3_2
T3_1
T3_3
B3_1
B3_2
B3_3
B0_3
B0_1
B0_2
B5_1
B5_3
T5_2
T5_1
B5_2
T5_3
T7_3
T7_1
T7_2
1
0.8
0.6
0.4
0.2
0


**Heatmap**
