# Supplementary material for: Transient acceleration of Lyophyllum decastes mycelial biomass accumulation by Acanthopanax senticosus extract
Source: Front Microbiol. 2026 Sep 3;17:1894250. doi: 10.3389/fmicb.2026.1894250 (PMC13582560; doi:10.3389/fmicb.2026.1894250)
Supplement: Supplementary file 6 [file Data_Sheet_6.pdf]

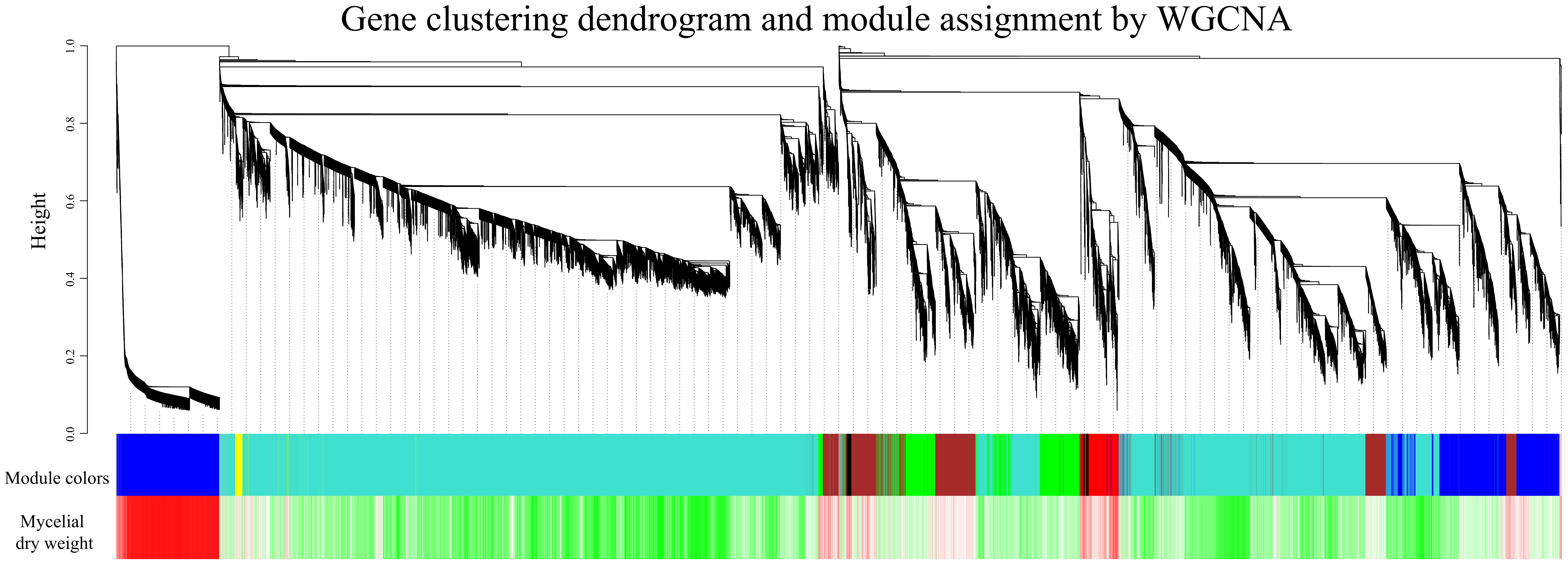

Gene clustering dendrogram and module assignment by WGCNA
Height
1.0
0.8
0.6
0.4
0.2
0.0
Module colors
Mycelial
dry weight
