# Supplementary material for: Transient acceleration of Lyophyllum decastes mycelial biomass accumulation by Acanthopanax senticosus extract
Source: Front Microbiol. 2026 Sep 3;17:1894250. doi: 10.3389/fmicb.2026.1894250 (PMC13582560; doi:10.3389/fmicb.2026.1894250)
Supplement: Supplementary file 7 [file Data_Sheet_7.pdf]

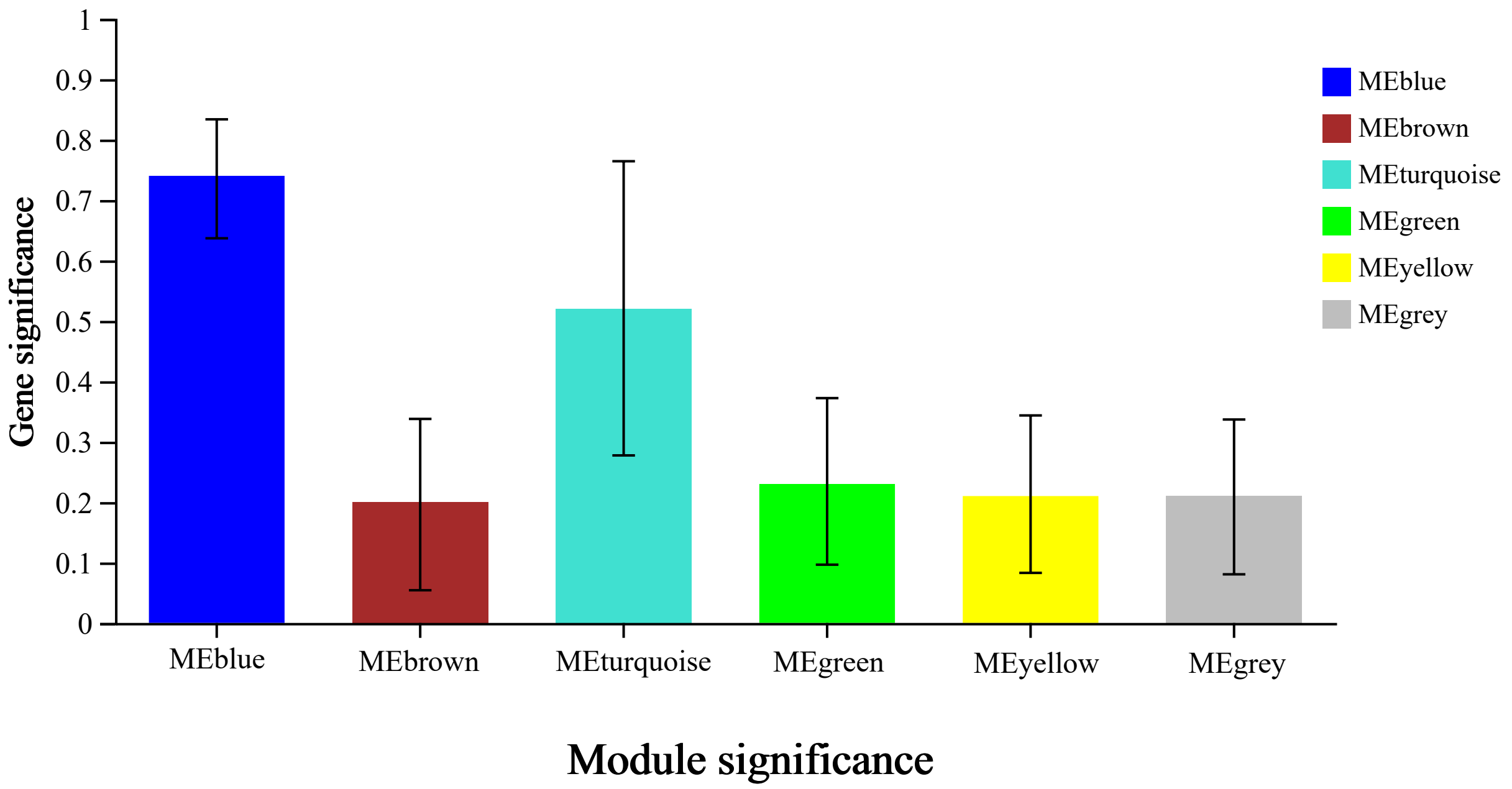

Gene significance
1
0.9
0.8
0.7
0.6
0.5
0.4
0.3
0.2
0.1
0
MEblue
MEbrown
MEturquoise
MEgreen
MEyellow
MEgrey
Module significance
MEblue
MEbrown
MEturquoise
MEgreen
MEyellow
MEgrey
