# Supplementary material for: Transient acceleration of Lyophyllum decastes mycelial biomass accumulation by Acanthopanax senticosus extract
Source: Front Microbiol. 2026 Sep 3;17:1894250. doi: 10.3389/fmicb.2026.1894250 (PMC13582560; doi:10.3389/fmicb.2026.1894250)
Supplement: Supplementary file 8 [file Data_Sheet_8.pdf]

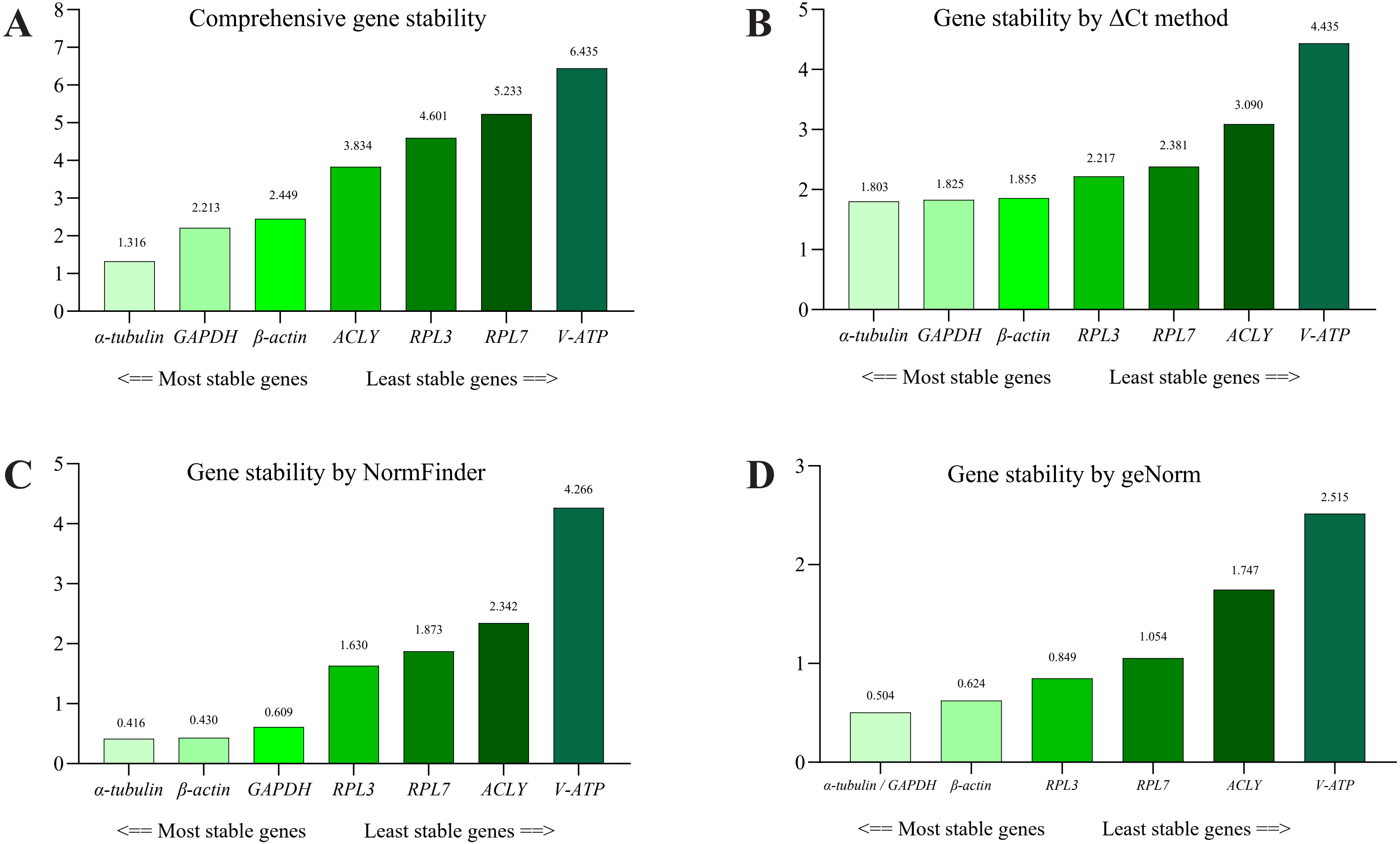
A
Comprehensive gene stability
8
7
6
5
4
3
2
1
0
1.316
2.213
2.449
3.834
4.601
5.233
6.435
α-tubulin
GAPDH
β-actin
ACLY
RPL3
RPL7
V-ATP
<== Most stable genes
Least stable genes ==>
B
Gene stability by ΔCt method
5
4
3
2
1
0
1.803
1.825
1.855
2.217
2.381
3.090
4.435
α-tubulin
GAPDH
β-actin
RPL3
RPL7
ACLY
V-ATP
<== Most stable genes
Least stable genes ==>
C
Gene stability by NormFinder
5
4
3
2
1
0
0.416
0.430
0.609
1.630
1.873
2.342
4.266
α-tubulin
β-actin
GAPDH
RPL3
RPL7
ACLY
V-ATP
<== Most stable genes
Least stable genes ==>
D
Gene stability by geNorm
3
2
1
0
0.504
0.624
0.849
1.054
1.747
2.515
α-tubulin / GAPDH
β-actin
RPL3
RPL7
ACLY
V-ATP
<== Most stable genes
Least stable genes ==>
